# Supplementary figures and images for: The ciliopathy protein CCDC66 controls mitotic progression and cytokinesis by promoting microtubule nucleation and organization
Source: PLoS Biol. 2022 Jul 18;20(7):e3001708. doi: 10.1371/journal.pbio.3001708 (PMC9333452; doi:10.1371/journal.pbio.3001708)

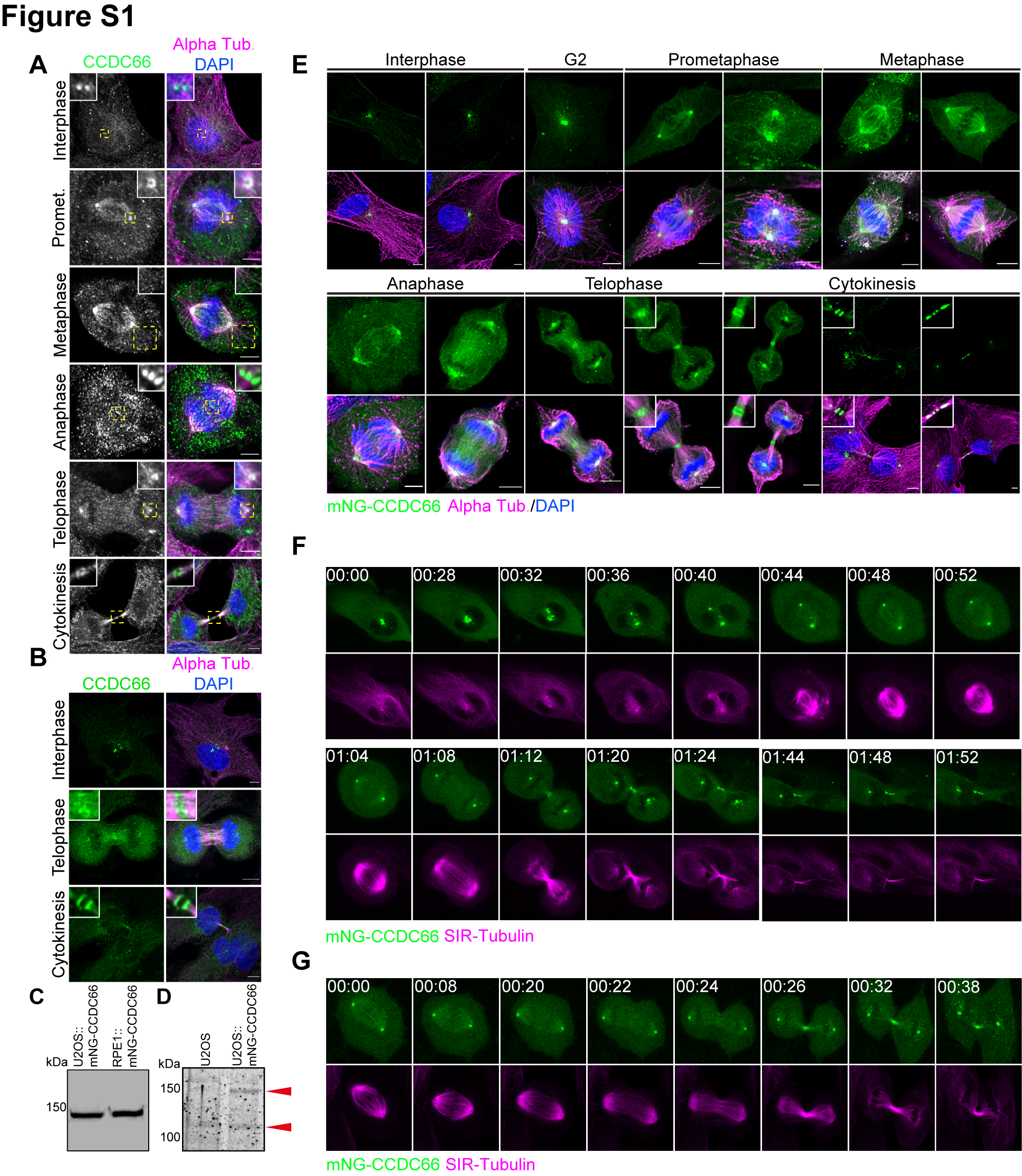

Supplement: S1 Fig — (A) Localization of CCDC66 at different stages of the cell cycle. U2OS were fixed with methanol followed by acetone and stained for CCDC66, alpha-tubulin, and DAPI. Scale bar: 5 μm, insets show 4× magnifications of the boxed regions. (B) Validation of RPE1::mNeonGreen-CCDC66 expression with antibody. RPE1::mNeonGreen-CCDC66 stable cell line was fixed with methanol and stained with CCDC66 antibody and alpha-tubulin. Insets show 4× zoom. Scale bar: 5 μm. (C) Validation of mNeonGreen-CCDC66 expression in U2OS::mNeonGreen-CCDC66 and RPE1::mNeonGreen-CCDC66 stable lines by immunoblotting. Extracts from cells were prepared, resolved by SDS-PAGE and blotted with mNeonGreen antibody. (D) Relative expression level of mNeonGreen-CCDC66 compared to endogenous protein in U2OS cells. Extracts from cells were prepared, resolved by SDS-PAGE and blotted with CCDC66 antibody. (E) Localization of mNeonGreen-CCDC66 at different stages of cell cycle. RPE1 cells stably expressing mNeonGreen-CCDC66 fusion (RPE1::mNeonGreen-CCDC66) were fixed with 4% PFA and stained for alpha-tubulin and DAPI. Scale bar: 5 μm. (F) Dynamic localization of mNeonGreen-CCDC66 throughout the cell cycle. U2OS cells stably expressing mNeonGreen-CCDC66 fusion (U2OS::mNG-CCDC66) were incubated with 100 nM SiR-Tubulin overnight. Images are acquired every 4 min using confocal microscopy. Shown are sixteen time-lapse images from S2 Movie at indicated time points to show dynamic localization of mNeonGreen-CCDC66 to spindle poles and microtubules during cell division. (G) Dynamic localization of mNG-CCDC66 throughout the cell cycle. RPE1::mNG-CCDC66 were incubated with 100 nM SiR-Tubulin overnight. Images were acquired every 2 min using confocal microscopy. Shown are 14 time-lapse images from S1 Movie at the indicated time points. CCDC66, coiled-coil domain-containing protein 66; SiR-tubulin, silicon rhodamine (SiR) tubulin. (TIF) [file pbio.3001708.s001.tif]

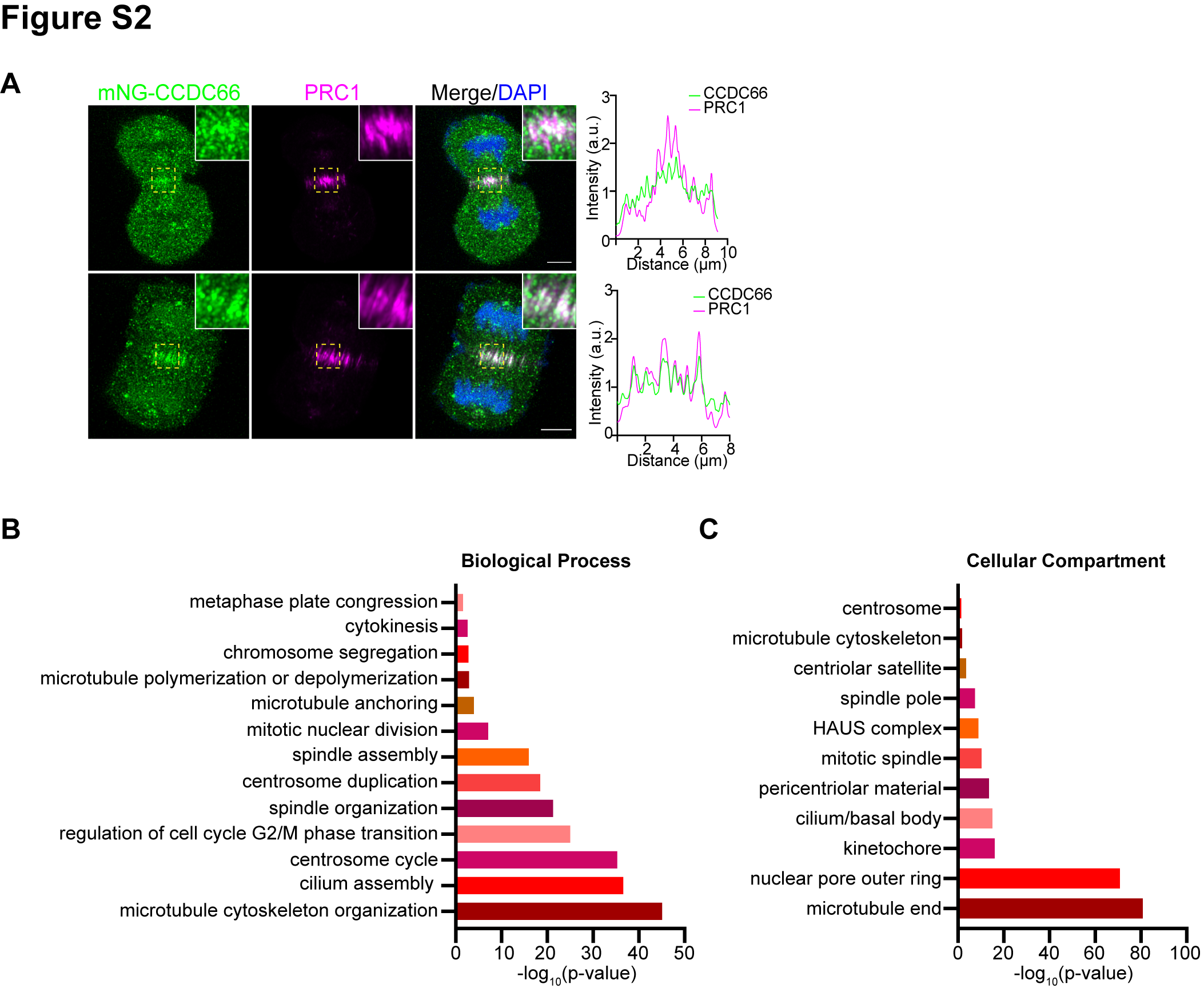

Supplement: S2 Fig — (A) Localization of mNeonGreen-CCDC66 in RPE1 cells relative to PRC1 during anaphase. RPE1::mNeonGreen-CCDC66 cells were fixed with 4% PFA and stained for PRC1 and DAPI. Graphs show the plot profiles to assess co-localization with the indicated marker. Using ImageJ, a straight line was drawn on the midbody, and intensity along the distance was plotted on Graphpad Prism. (B, C) GO-enrichment analysis of the CCDC66 proximity interactors based on their (B) biological process and (C) cellular compartment. The x-axis represents the log-transformed p-value (Fisher’s exact test) of GO terms. The data underlying the graphs showing the plot profiles in the figure can be found in S1 Data. CCDC66, coiled-coil domain-containing protein 66; PFA, paraformaldehyde; PRC1, protein regulator of cytokinesis 1; DAPI, 4′,6-diamidino-2-phenylindole. (TIF) [file pbio.3001708.s002.tif]

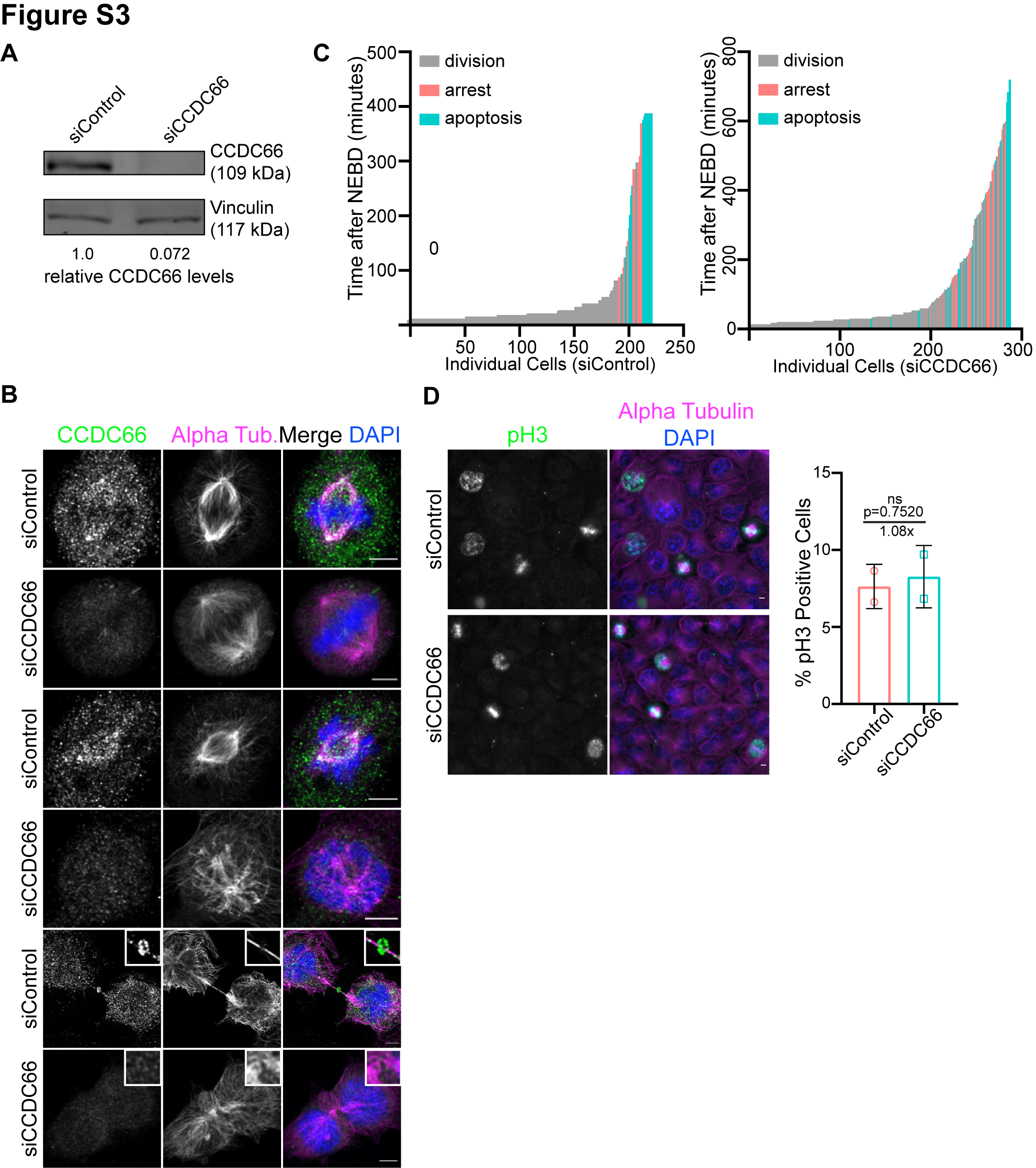

Supplement: S3 Fig — (A) Validation of the efficiency of RNAi-mediated CCDC66 depletion by western blotting and immunofluorescence. U2OS cells were transfected with siControl or siCCDC66. Approximately 48 h after post-transfection, cells were fixed and stained with the indicated antibodies. In parallel, cell extracts immunoblotted for CCDC66 and vinculin (loading control). Band intensities were measured on ImageJ and normalized against background and vinculin intensities. Arbitrary value is determined based on siControl. (B) Validation of the CCDC66 antibody by immunofluorescence. U2OS cells were transfected with control or CCDC66 siRNA, fixed with methanol followed by acetone 48 h post-transfection and stained for CCDC66 and alpha-tubulin. Representative images are shown at different stages of the cell cycle to indicate the decrease in the signal of CCDC66 upon siCCDC66 transfection. Insets show 4× zoom of boxed areas. Scale bar: 5 μm. (C) Quantification of Fig 3A. The fate of individual cells was plotted as vertical bars, where the height of the bar represents the mitotic time, and the color of the bars represent the different fates including successful division (gray), mitotic arrest (pink), and apoptosis (cyan); n > 200 cells from each condition was quantified per condition. (D) Effect of CCDC66 depletion on mitotic index. U2OS cells were transfected with control or CCDC66 siRNA, fixed with methanol 48 h post-transfection and stained for the mitotic marker phospho-Histone3 (pH3), alpha-tubulin, and DAPI. Mitotic cells are counted based on DNA staining. Data represent the mean ± SEM of 2 independent experiments and n > 1,000 for all experiments. Mean mitotic cell number for siControl is 10.31 and mean mitotic cell number for siCCDC66 is 12.49. Representative images are shown. Scale bar: 5 μm. The data underlying the graphs shown in the figure can be found in S1 Data. CCDC66, coiled-coil domain-containing protein 66; siRNA, small interfering RNA; SEM, standard error of mean; DAPI, 4′, [file pbio.3001708.s003.tif]

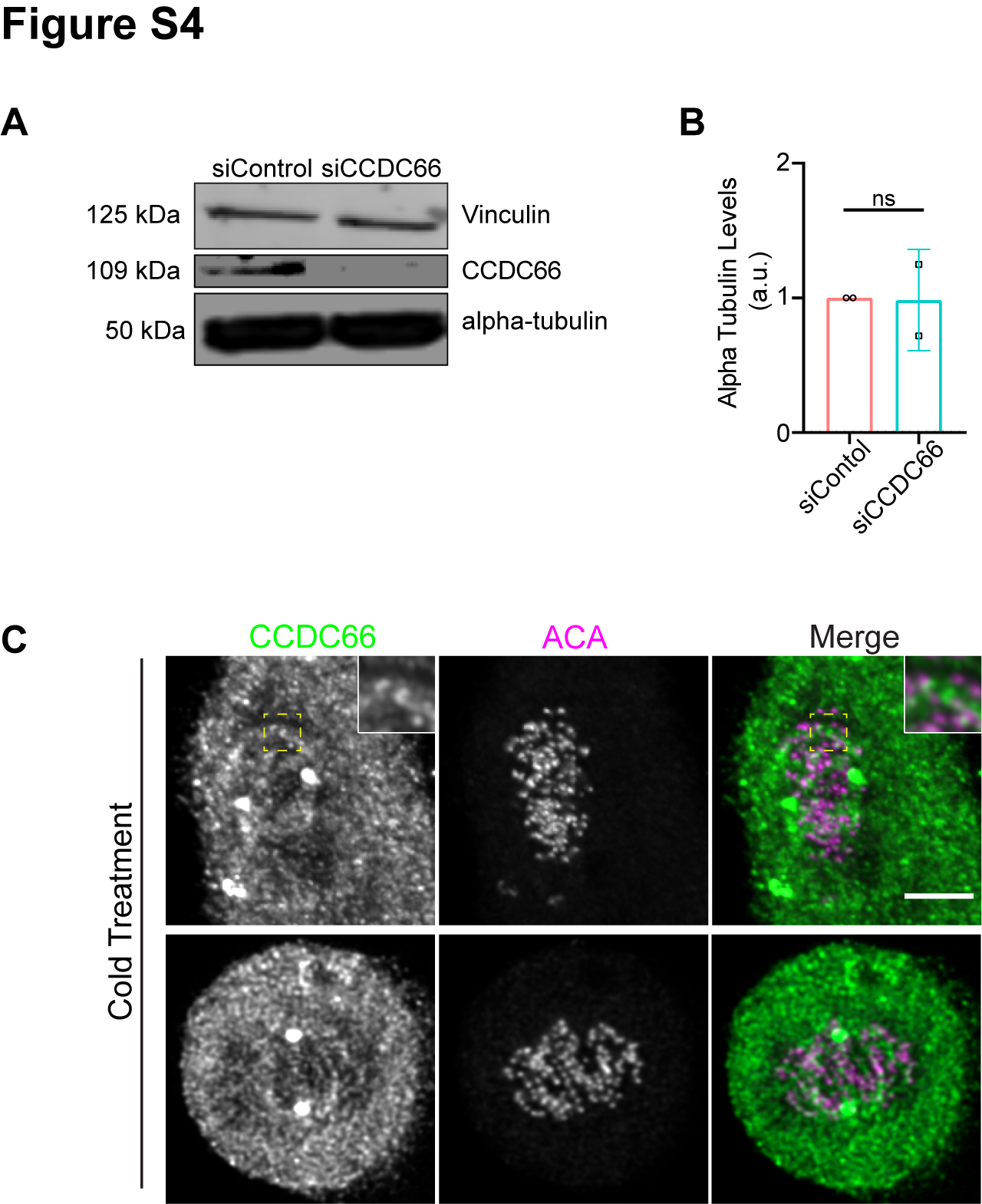

Supplement: S4 Fig — (A) Cellular abundance of tubulin in control and CCDC66-depleted cells. Cells are transfected with either control or CCDC66 siRNA. After 48 h, the lysates are collected and immunoblotted for alpha-tubulin, CCDC66, and vinculin (loading control). (B) Quantification of (A). Data represent mean ± SEM of 2 independent experiments. (ns: not significant). (C) CCDC66 localizes on K-fibers. U2OS cells were grown on coverslips and incubated in ice for 10 min before fixation with methanol followed by acetone. Cells were stained for CCDC66 and ACA. Inset shows 4× zoom of boxed area. Scale bar: 5 μm. The data underlying the graphs shown in the figure can be found in S1 Data. CCDC66, coiled-coil domain-containing protein 66; siRNA, small interfering RNA; SEM, standard error of mean; K-fiber, kinetochore fiber. (TIF) [file pbio.3001708.s004.tif]

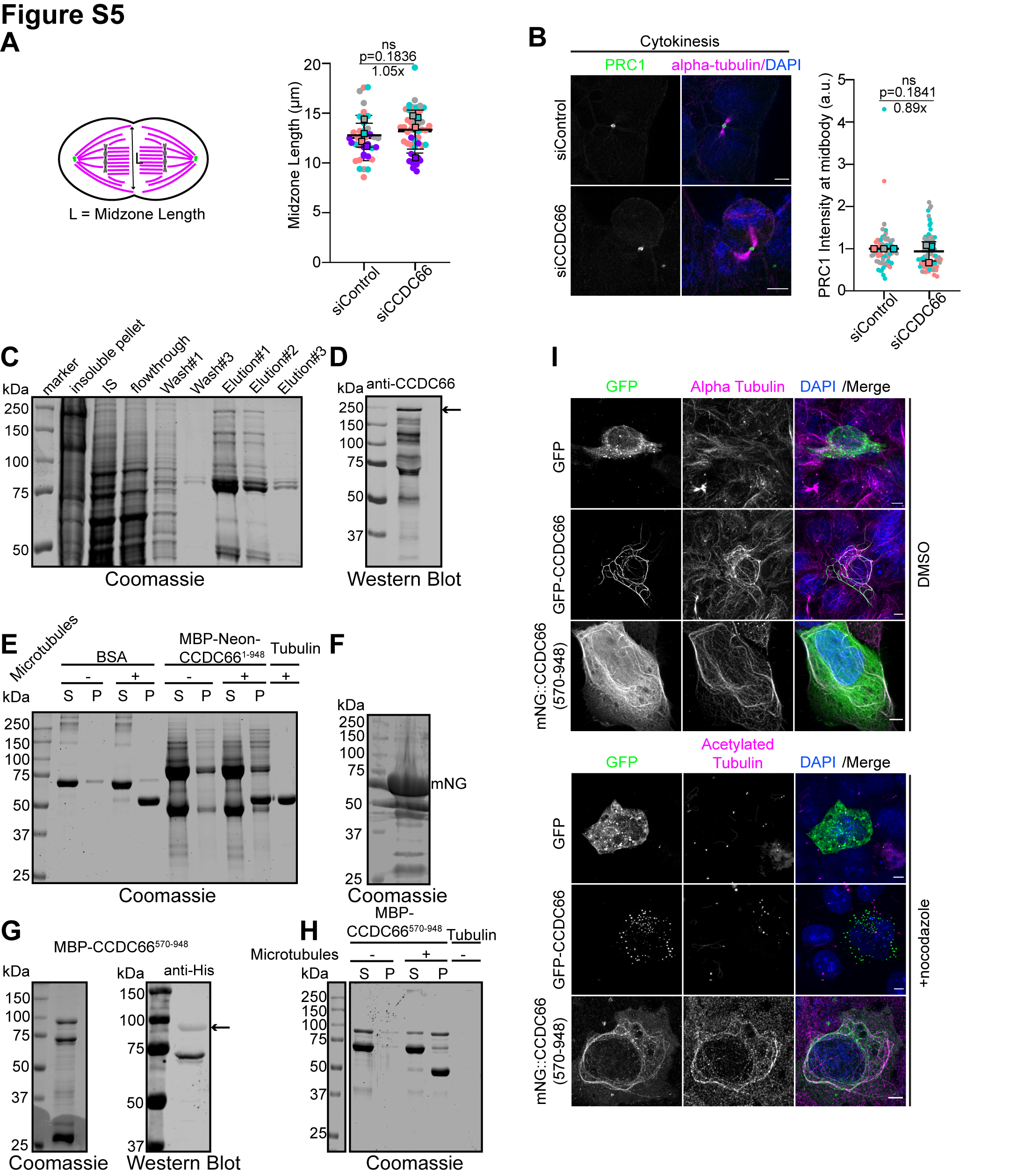

Supplement: S5 Fig — (A) Spindle midzone length is not altered by CCDC66 depletion. U2OS cells were transfected with control or CCDC66 siRNA, fixed with methanol followed 48 h post-transfection and stained for alpha-tubulin and DAPI. As shown in the representation, midzone length was measured as the distance between the most distant microtubule ends in the midzone. Data represent the mean ± SEM of 4 independent experiments. (ns: not significant). (B) CCDC66 depletion does not alter PRC1 midbody levels. U2OS cells were transfected with siRNA then fixed with methanol after 48 h and stained for PRC1, alpha-tubulin, and DAPI. Images represent cells from anaphase and cytokinesis. Scale bar: 5 μm. For quantification, PRC1 intensity was measured on ImageJ, the background signal was subtracted, and normalized value was multiplied with area. Arbitrary value was determined by normalizing against siControl. (ns: not significant). (C) His-MBP-mNeonGreen-CCDC66 purification. His-MBP-mNeonGreen-CCDC66 was purified from insect cells using Ni-NTA agarose beads. Coomassie staining shows the proteins in pellet, initial sample, flowthrough, wash, and elutions. (D) Validation of His-MBP-mNeonGreen-CCDC66 purification. Purified His-MBP-mNeonGreen-CCDC66 purification was run on SDS-PAGE and blotted with CCDC66 antibody. Arrow corresponds to the full-length His-MBP-mNeonGreen-CCDC66. (E) His-MBP-mNeonGreen-CCDC66 directly interacts with microtubules. His-MBP-mNeonGreen-CCDC66 was purified from insect cells, and in vitro microtubule pelleting was performed and visualized by Coomassie staining. BSA was used as negative control. S stands for supernatant, P stands for pellet. (F) Validation of MBP-mNeonGreen purification with Coomassie. (G) Validation of MBP-CCDC66 (570–948) purification with Coomassie. MBP-His-CCDC66 (570–948) was purified from bacterial culture using Ni-NTA agarose beads. Purified protein was run on SDS-PAGE. Coomassie staining and western blotting with anti-His antibody shows the purified prot [file pbio.3001708.s005.tif]

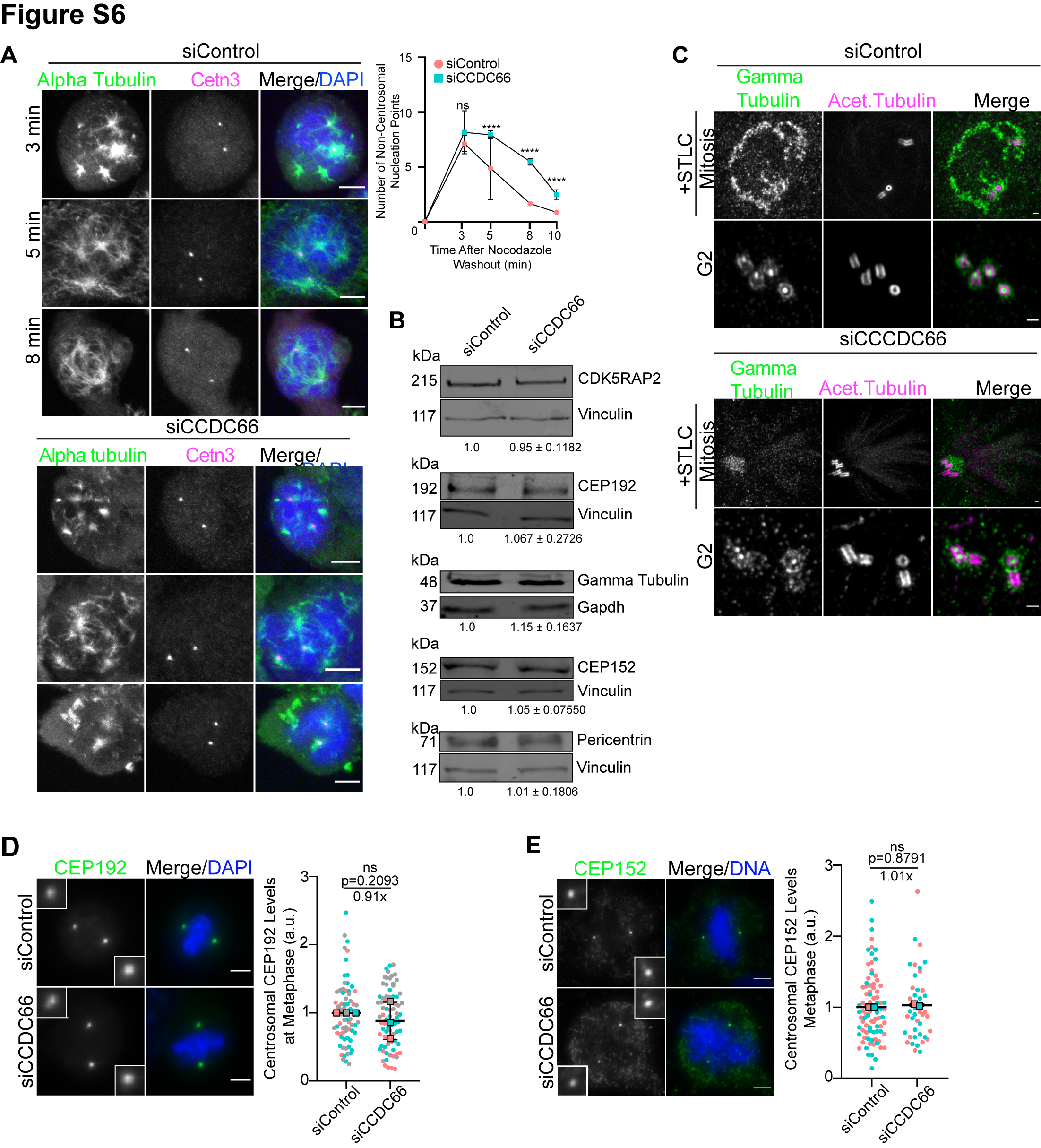

Supplement: S6 Fig — (A) Quantification of microtubule nucleation sites following nocodazole washout of STLC-synchronized control and CCDC66-depleted cells. Representative images are shown for control and CCDC66-depleted cells. The graph indicates the number of microtubule nucleation points that are counted from Centrin 3 and alpha-tubulin signals. Data represent the mean ± SEM of 2 independent experiments. (**p < 0.01) Scale bar: 5 μm. (B) Effect of CCDC66 depletion on the cellular abundance of PCM proteins. U2OS cells were transfected with siControl or siCCDC66, and 48 h after transfection extracts from cells were immunoblotted for CDK5RAP2, CEP192, CEP152, gamma-tubulin, pericentrin and vinculin (loading control), or GAPDH (loading control). Band intensities were measured on ImageJ and normalized against background and vinculin intensities. Data represent the mean ± SEM of 3 independent experiments. (C) U-ExM analysis of control and CCDC66 depleted cells. U2OS cells were transfected with control and CCDC66 siRNA. Approximately 48 h post-transfection, cells were synchronized by 16 h STLC treatment and prepared for imaging. Cells were stained for gamma-tubulin and acetylated tubulin, imaged using confocal microscopy and deconvolved. Mitotic and G2 cells were picked for representation. (D, E) Effects of CCDC66 depletion on abundance of PCM proteins at the centrosomes. U2OS cells were transfected with control and CCDC66 siRNA. After 48 h, cells were fixed with methanol and stained for (D) CEP192 and (E) CEP152. Centrosomal abundance of PCM proteins was measured as described in Fig 4D. Images for each panel represent cells captured with the same camera settings from the same coverslip. Data represent mean ± SEM of 2 (CEP152) or 3 (CEP192) independent experiments. (ns: not significant). Scale bar: 5 μm. The data underlying the graphs shown in the figure can be found in S1 Data. CDK5RAP2, CDK5 regulatory subunit-associated protein 2; CEP152, centrosomal protein of 152 kDa; CEP192, centrosom [file pbio.3001708.s006.tif]

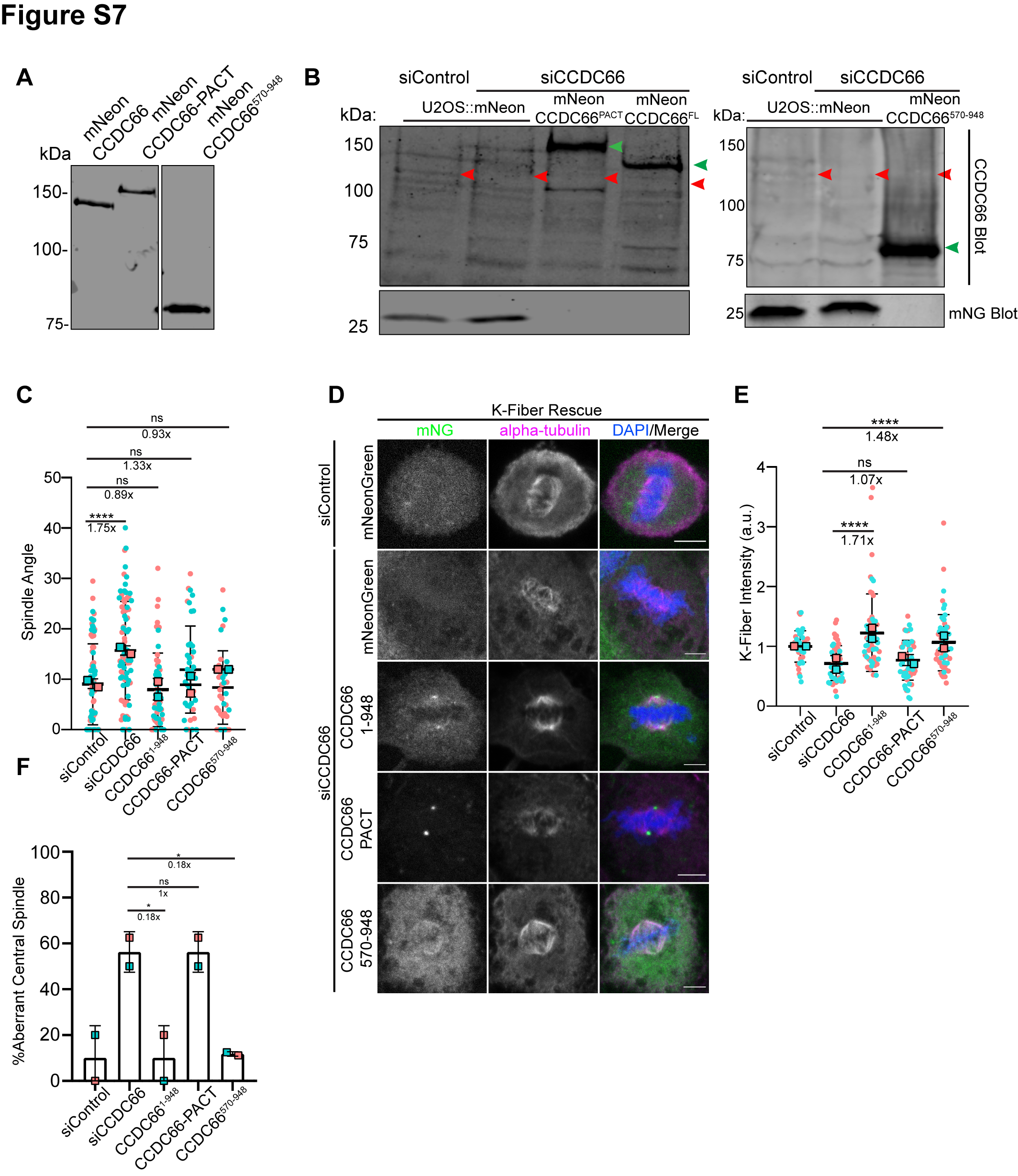

Supplement: S7 Fig — (A) Validation of U2OS cells lines that stably express siRNA-resistant mNeonGreen-CCDC66, mNeonGreen-CCDC66-PACT, and mNeonGreen-CCDC66 (570–948). Extracts from cells were prepared, resolved by SDS-PAGE, and blotted with mNeonGreen antibody. (B) Validation of siRNA resistance of the CCDC66 rescue constructs. U2OS cells were transfected with control and CCDC66 siRNA. Approximately 48 h post-transfection extracts from cells were prepared, resolved by SDS-PAGE, and blotted with CCDC66 antibody. The red arrowheads indicate endogenous CCDC66, which is masked due to higher expression of the fusion proteins and high background associated with the CCDC66 antibody. The green arrowheads indicate the mNeonGreen fusions of CCDC66. (C) Quantification of Fig 7A. Spindle angle was calculated by the formula α = 180*tan−1(h/L)/π where h represents the stack difference between 2 centrosomes, L represents the distance between centrosomes when projected onto 1 z plane. Data represent the mean ± SEM of 2 independent experiments. (**p < 0.01 ***p < 0.001 ****p < 0.0001). (D) Representative images for the K-fiber rescue experiment performed using U2OS::mNeonGreen, U2OS::mNeonGreen-CCDC661-948, U2OS::mNeonGreen-CCDC66-PACT, and U2OS::mNeonGreen-CCDC66570-948 stable cells. Cells were transfected with control and CCDC66 siRNA. Approximately 48 h post-transfection, they were fixed with methanol and stained for alpha-tubulin and DAPI. Scale bar: 5 μm. (E) Quantification of Fig 7D. Graph represents the intensity of K-fibers with mean ± SEM of 2 independent experiments. (****p < 0.0001, ns: not significant). (F) Quantification of Fig 7D. Graph represents the percentage of aberrant central spindle with mean ± SEM of 2 independent experiments. (****p < 0.0001, ns: not significant). The data underlying the graphs shown in the figure can be found in S1 Data. CCDC66, coiled-coil domain-containing protein 66; K-fiber, kinetochore fiber; siRNA, small interfering RNA; SEM, standard error of mean. (TIF) [file pbio.3001708.s007.tif]
