# Supplementary material for: The ciliopathy protein CCDC66 controls mitotic progression and cytokinesis by promoting microtubule nucleation and organization
Source: PLoS Biol. 2022 Jul 18;20(7):e3001708. doi: 10.1371/journal.pbio.3001708 (PMC9333452; doi:10.1371/journal.pbio.3001708)

Figure S1C

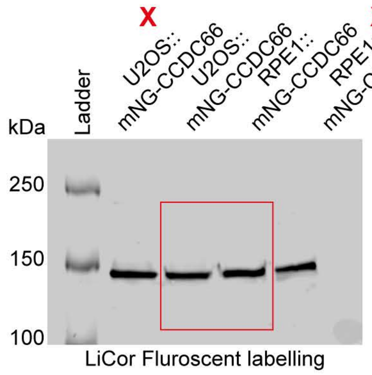

Figure S1D

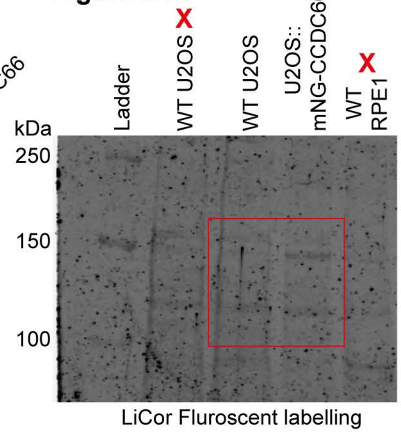

Figure 2E

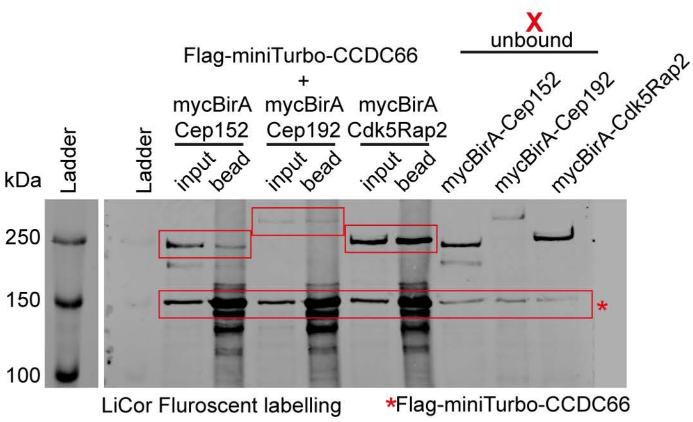

Figure 2E

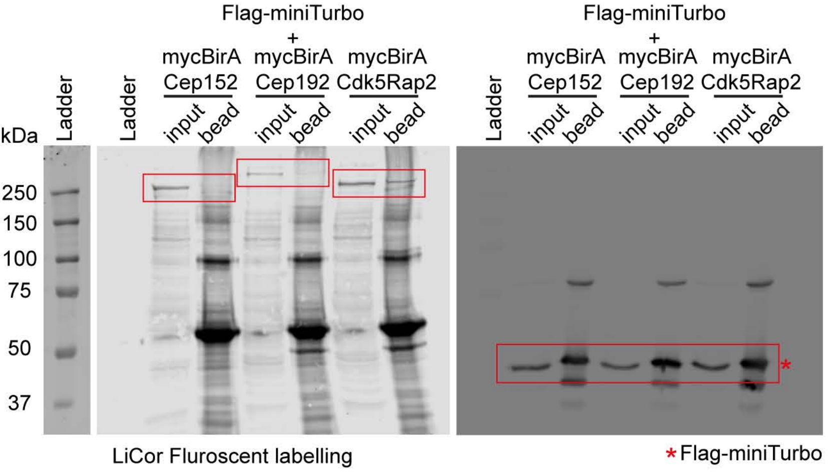

Figure 2E

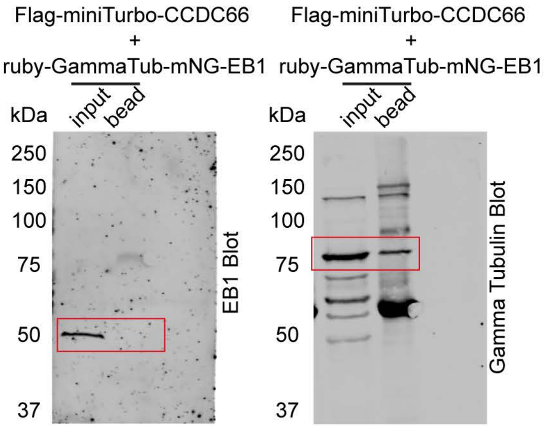

Figure 2E

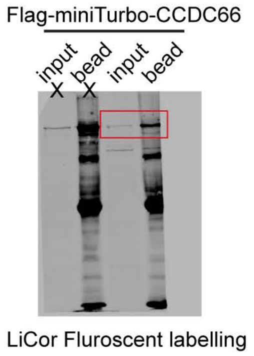

Figure 2F

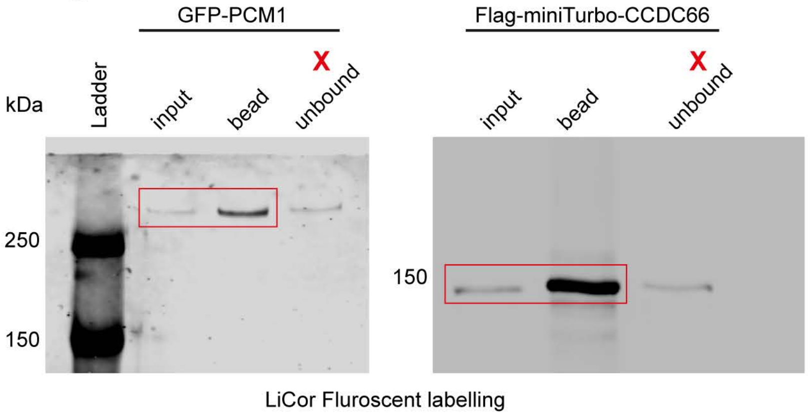

Figure 2F

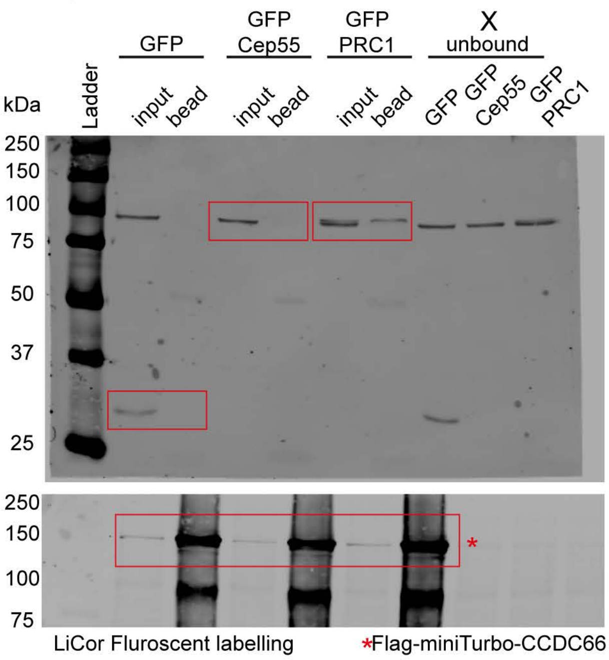

Figure S3A

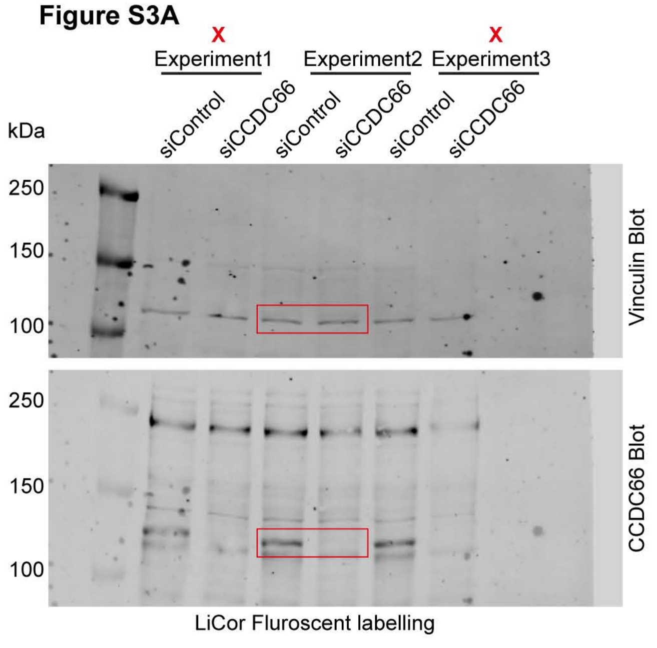

Figure S4A

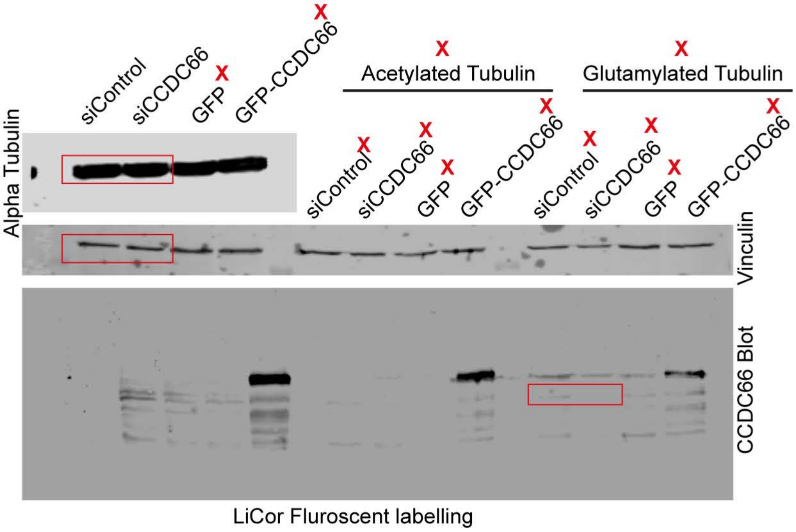

Figure S5C

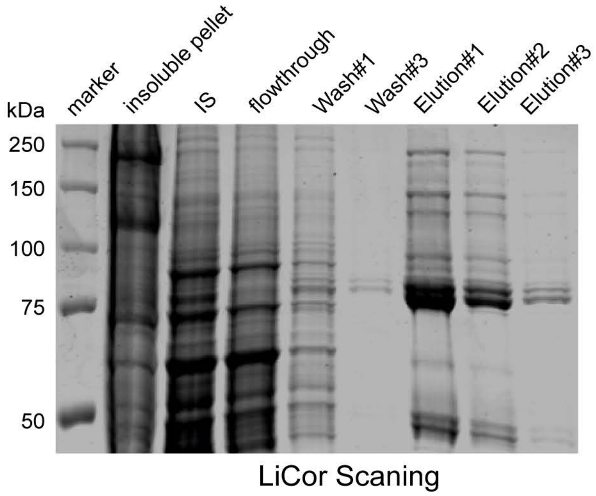

Figure S5D

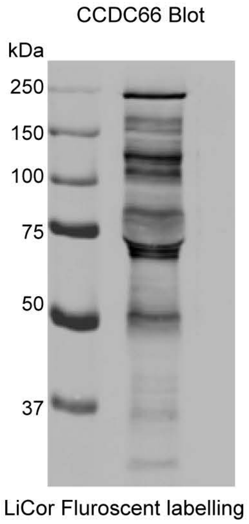

Figure S5E

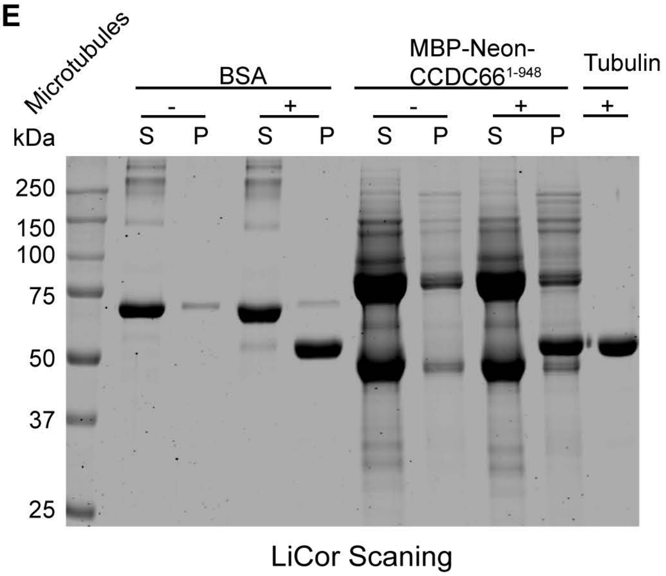

Figure S5F

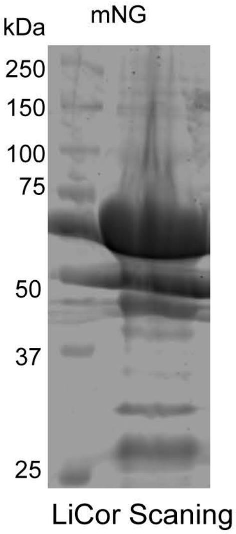

Figure S5G

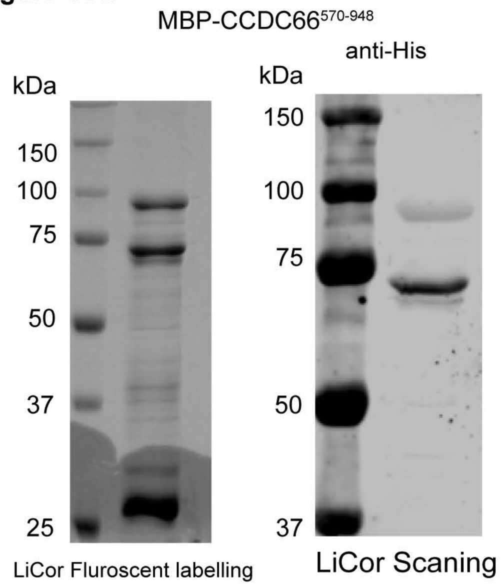

Figure S5H

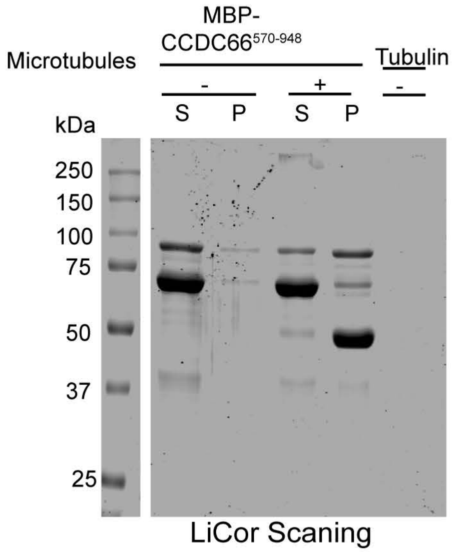

Figure S6B

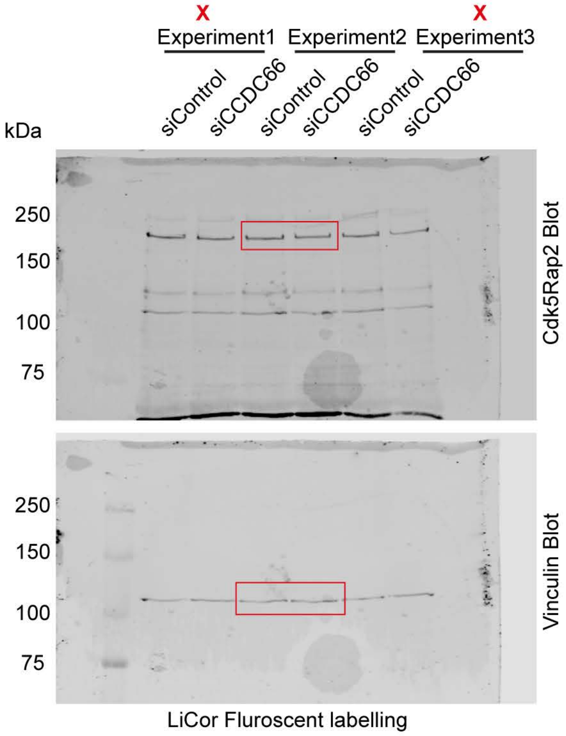

Figure S6B

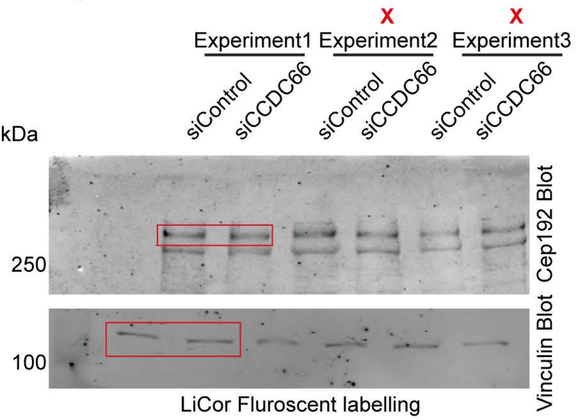

Figure S6B

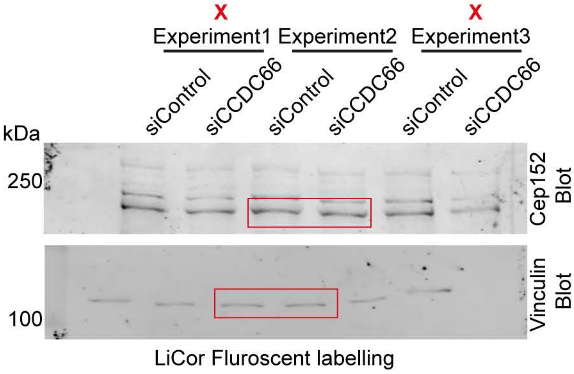

Figure S6B

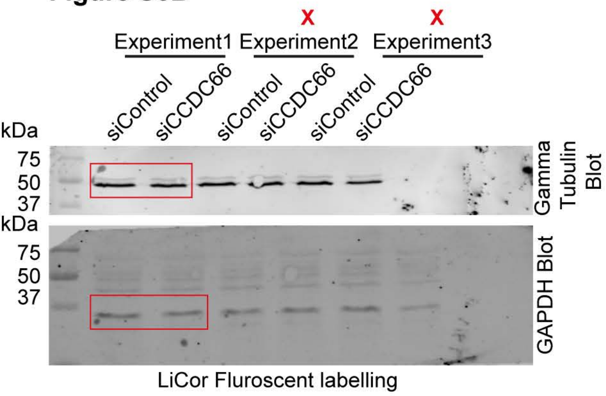

Figure S6B

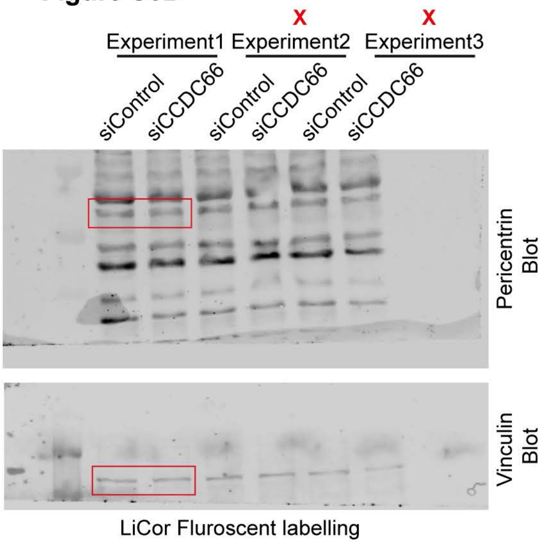

Figure S7A

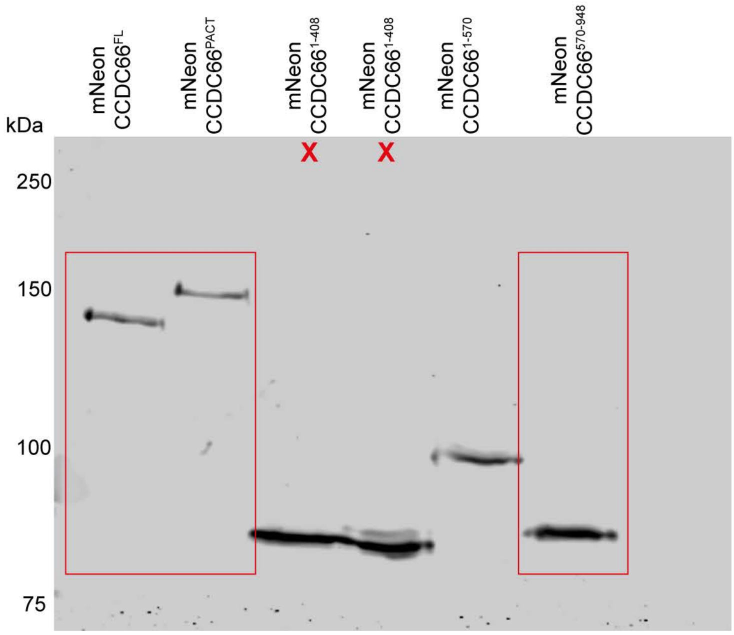

Figure S7B

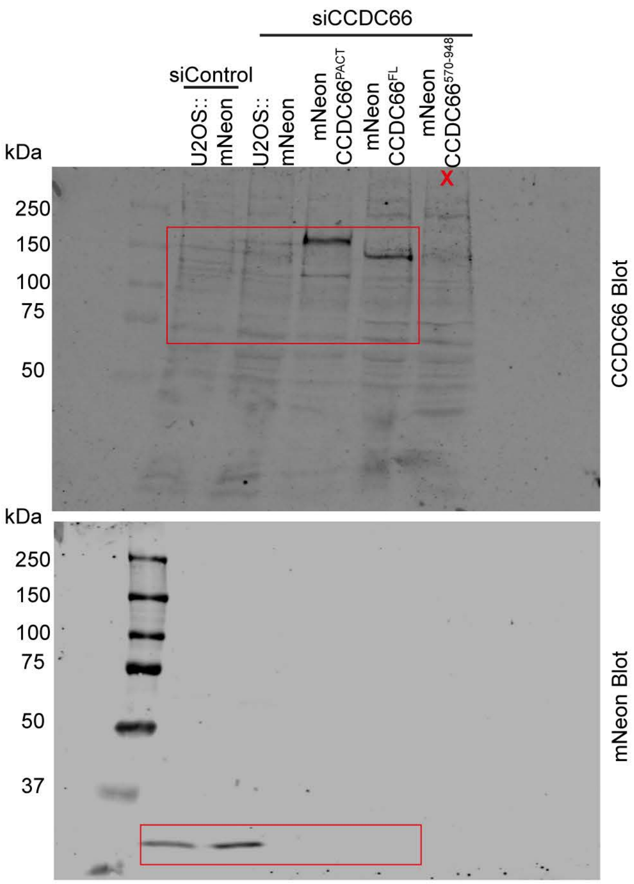

Figure S7B

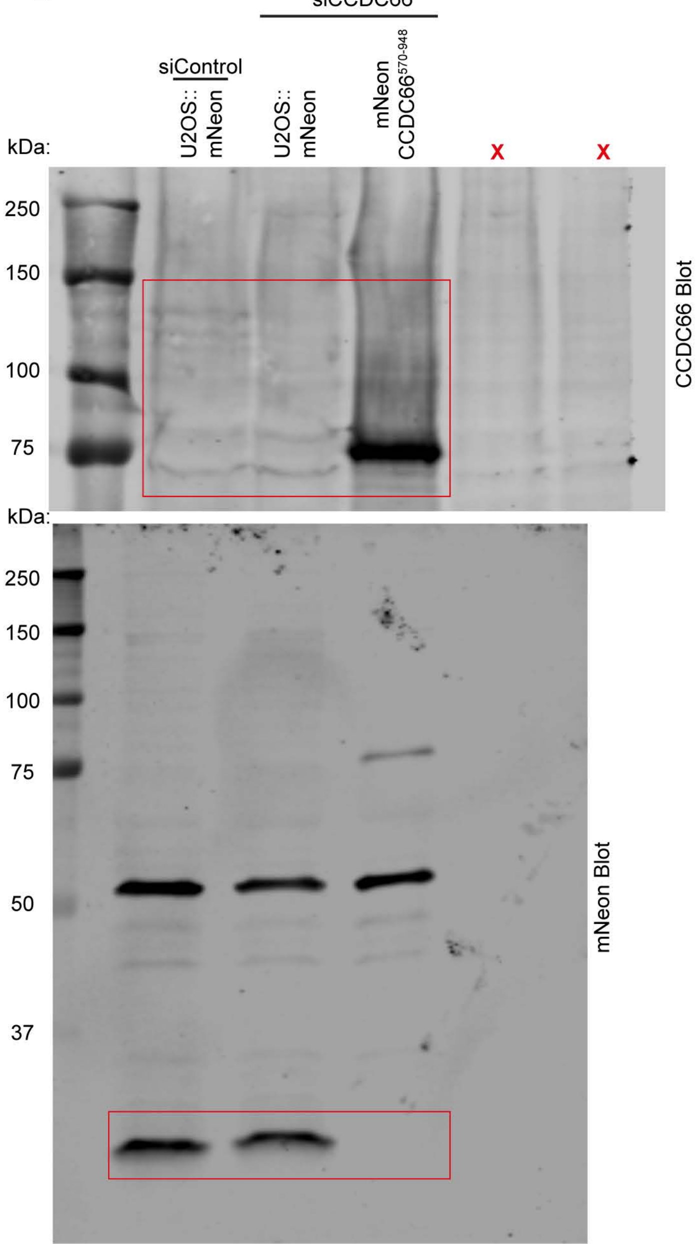

Supplement: S1 Raw Images — Red boxes are used to show the represented blot in the figures. Red crosses show the parts of the blot that are not used in the figures. (PDF) [file pbio.3001708.s008.pdf]
