# Supplementary material for: The ciliopathy protein CCDC66 controls mitotic progression and cytokinesis by promoting microtubule nucleation and organization
Source: PLoS Biol. 2022 Jul 18;20(7):e3001708. doi: 10.1371/journal.pbio.3001708 (PMC9333452; doi:10.1371/journal.pbio.3001708)
Supplement: Attachment — Submitted filename: PointByPointResponse_final.pdf [file pbio.3001708.s019.pdf]

Reviewer #1

The manuscript by Batman et al. presents a functional analysis of the microtubule-associated protein CCDC66 in cycling cells, where the authors identify roles in mitotic spindle assembly and cytokinesis. Previous work by the same group characterized CCDC66 as a protein with roles in cilia in quiescent cells. The work is quite a comprehensive study that, surprisingly, finds involvements of CCDC66 in a range of different processes. The study is potentially interesting to the centrosome and cilia communities.

We thank the reviewer for the constructive criticism of our manuscript. We are encouraged to see that the reviewer found our study comprehensive and of interest to centrosome and cilia communities.

A major issue with the work is technical, the inability to detect endogenous CCDC66. For this reason, the authors rely on the detection of tagged CCDC66 for all of their localization studies. However, rather than using endogenous tagging, they employ expression by viral transduction with potential overexpression of CCDC66. This raises the question of how relevant the multiple localizations are that they observe. Apart from this, there are several experimental issues such as lack of controls etc. Overall, the manuscript has potential but will require significant revision.

We agree with the reviewer's concern on whether mNG-CCDC66 localizatoin in stable lines reflect endogenous localization and lack of controls in several experiments. We addressed the major concerns regarding endogenous localization of CCDC66 during cell division and inclusion of controls for *in vitro* and cellular assays and developed a strategy to address the remaining concerns. As detailed below, the results of revision experiments collectively strengthened our conclusions on the role of CCDC66 during mitosis and cytokinesis.

Main points:

1) I am concerned that some of the multiple localizations are caused by overexpression. CCDC66 is a MAP and as such seems to bind to all microtubule structures including all interphase microtubules (e.g. S1D). The authors need to show the levels of expression of tagged CCDC66 relative to endogenous CCDC66 by western in Fig S1 using anti-CCDC66 antibody. Parent cell lines and an independent loading control should be included to compare levels between cell lines, since endogenous expression could be affected by the recombinant expression.

2) Related to point 1 above, if tagged CCDC66 levels in stable lines turns out to be significantly higher than endogenous levels, and if endogenous tagging cannot be provided, expression levels may be reduced by promoter truncation or FACS sorting. Otherwise it will be difficult to conclude any specific localizations of CCDC66. This in turn would also challenge current conclusions about the involvement of CCDC66 in the formation or function of certain microtubule-based structures, which are based on the localization studies.
We agree with the reviewer's major concern, which was raised by all three referees. Below are new results that together address this concern and confirms localization of CCDC66 to spindle poles and multiple microtubule-based structures during cell division:

- For the first submission, we tested three antibodies raised against different CCDC66 antigens in localization experiments (Bethyl Laboratories A303-339A - antigen: 898-948 a.a; Sigma SAB1408484 - antigen: 1-831 a.a.; Home-made = antigen: 570-948 a.a.) Although we optimized antibody concentration in methanol, PFA or methanol/PFA-fixed U2OS cells, we had not observed consistent endogenous localization to mitotic structures. This is why we only included localization data of cells stably expressing CCDC66 fusion protein.

  For the revisions, we worked on further optimization of immunofluorescence protocols (fixation, blocking and antibody dilution steps) with the Bethyl antibody that we previously detected spindle pole and microtubule staining in RPE1 cells (Conkar et al. 2017). In U2OS cells fixed with methanol-acetone, Bethyl antibody generated consistent results to demonstrate co-localization of endogenous CCDC66 to the spindle poles and microtubule-based structures of cell division (bipolar spindle, central spindle, midbody) despite high cytoplasmic and punctate background

(Fig. 1A and S1A). Using U2OS cells depleted for CCDC66, we also confirmed the specificity of the antibody in detecting the mitotic and cytokinetic structures (Fig. S3B)

- We examined localization of GFP-CCDC66 using a previously characterized RPE1::GFP-CCDC66 cell line that expresses near-endogenous levels of GFP-CCDC66 (Conkar et al. JCS 2017). Like mNG-CCDC66 localization in stable lines, GFP-CCDC66 also localizes to the bipolar spindle in metaphase and midbody in cytokinesis. We note that we used mNeonGreen-CCDC66 in our manuscript as its brightness and photostability is advantageous for live imaging experiments.

- We used lentivirus infection followed by puromycin selection to generate U2OS::mNG-CCDC66 and RPE1::mNG-CCDC66 stable lines. We prepared lentivirus using an expression vector that has EF1 promoter (pCDH-EF1-MCS-T2A-Puro). Using CCDC66 antibody, we examined expression levels of mNeonGreen-CCDC66 in U2OS stable lines. Despite the punctate background, the western blot data demonstrates that mNeonGreen-CCDC66 is expressed at near-endogenous levels in the U2OS stable line (Fig. S1D) . We will also quantify mNG-CCDC66 expression levels relative to endogenous protein in RPE1::mNG-CCDC66 cell line.

Taken together, these results show that CCDC66 localizes to spindle poles and multiple microtubule-based structures during cell division and confirms that mNeonGreen-CCDC66 localizes similar to the endogenous protein.

3) Fig. 2C: Contrary to the claim in the text the cells are not mitotic. The nuclear envelope seems intact based on DAPI, so at the very best they are in G2. In the first nocodazole-treated example the gamma-tubulin staining is much weaker, as would be expected for an interphase cell. In the other examples in 2C the centrosome signal of gamma-tubulin looks much stronger, which could indicate G2.
We agree with the reviewer that the presented images in Fig. 2C for centrosome localization of control and nocodazole-treated cells represent G2 cells. We revised the sentence in the revised manuscript as follows:
"Notably, the centrosome-associated pool of CCDC66 was maintained in nocodazole-treated cells, confirming that this pool binds to centrosomes independent of MTs (Fig. 2C)."

3) Fig. 2D: In some of the examples the staining during cytokinesis shows signals outside the PRC1 region, which is not commented on. Is this stage-dependent, is it seen in all cells?
As the reviewer highlighted, we also noted the accumulation of mNG-CCDC66 outside the PRC1 ring during cytokinesis. This staining in the first two panels of Fig. 2D was only prominent in cells at later stages of cytokinesis, which is nicely represented in Movie 1 and Movie S1. To define this structure, we performed co-localization experiments of mNG_CCDC66 with known midbody markers (Fig. 2D). However, we did not detect any co-localization with any of the tested markers neither did we find a report of a similar structure in literature. This might be a midbody structure specific to CCDC66, which we discussed in the revised manuscript by including the following sentence:

"Of note, we observed accumulation of CCDC66 outside the midbody marked by PRC1 and Kif23 in cells at later stages of cytokinesis, which is also evident in the dynamic behavior of mNG-CCDC66 during cell division (Fig. 2D, Movie 1, Movie S1)."

4) The Co-IPs in Fig. 2E and F cannot be presented and interpreted in this manner. The way it is described, it seems the authors have coexpressed pairs of Flag-CCDC66 with various myc-tagged proteins. If so, for each pair Flag-CCDC66 needs to be shown in input/IP lanes. Moreover, for each interaction a control is needed such as coexpression of the respective myc-tagged protein with a Flag-tagged control protein, followed by Flag IP. As currently presented, all of the interactions could be unspecific binding to the anti-Flag beads. The fact that some proteins cannot be detected in the IP lane does not change that.
- We did run blots to confirm FLAG-miniTurbo-CCDC66 pulldown for each interactor we tested. Since immunoblotting with FLAG antibodies confirmed efficient pulldown of FLAG-miniTurbo-CCDC66 in all

cases, we included one representative pulldown in Fig. 2F. We will include the rest of the blots in the figures that include the uncropped raw data for Fig. 2F (shown below).

- As pointed out by the reviewer, whether the selected centrosome proteins nonspecifically binds to Flag-miniTurbo or not is also an important control, which we now included in Fig. 2E. Unlike FLAG-miniTurbo-CCDC66, FLAG-miniTurbo did not interact with myc-BirA* fusions of CDK5RAP2, CEP152 and CEP192. We note that there are two bands in CDK5RAP2 in eluates that do not correspond to the expected size of myc-BirA*-CDK5RAP2 and is not as enriched as it is when FLAG-miniTurbo-CCDC66 is used as a bait. To ensure this is the case and this interaction is specific, we will repeat the pulldown experiment for CDK5RAP2.

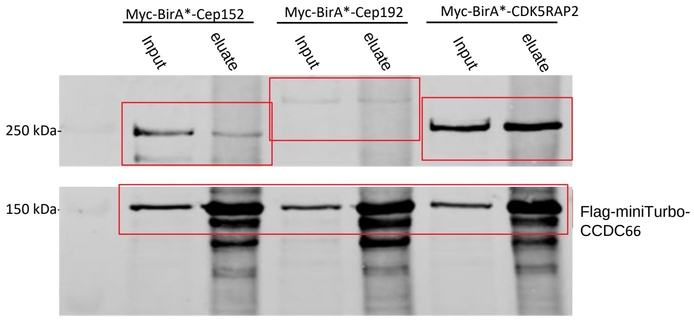

5) Fig. 3B: Considering the distribution of data points, I am surprised by the small p value. Was n=3 used here? (please also check for the middle graph in 4A). Also, this data is clearly not showing normal distribution.

We performed three independent experiments to assess the outcome of depletion on mitotic progression using live imaging (Fig. 3) and on spindle properties (Fig. 3). We visualized the data points for each experimental replicate by different colors (pink, cyan, grey) and used two-tailed unpaired t-test to calculate the statistical significance. As the reviewer pointed out, data in Fig. 3B do not follow the normal distribution. Therefore, we now applied a nonparametric alternative to t-test, Mann Whitney test, and calculated statistical significance of W = 14118, p<0.0001. We changed the figure legend and material and methods accordingly.

Data in the middle graph in Fig. 4A, which is the measurement of the spindle length, follows a normal distribution. Based on reviewer #3 comments, we will correct the spindle length measurement taking into account the tilting of the spindles in CCDC66 depletion, and applying the formula D=sqareroot (h^2L^2), which will still produce data points with normal distribution.

6) Fig. 4D: The conclusion that CCDC66 is a k-fiber protein is not warranted considering that its expression levels are unclear and that it basically binds all cellular microtubules.

As we addressed in point 1 to the reviewer's comment, we performed immunofluorescence experiments to assess whether CCDC66 localizes to k-fibers in cold-treated U2OS cells using the protocols we optimized for CCDC66 staining. Despite the high cytoplasmic and punctate background, CCDC66 antibody detected microtubules that associated with the centromeres as marked by ACA (Fig. S4C). The affinity is not as prominent as the one we observed using cells stably expressing mNG-CCDC66, which is likely due to increased abundance of CCDC66 in the stable line (Fig. 4E). Taken together, these results supports k-fiber localization of endogenous CCDC66.

7) Fig. 5: The description of the regrowth experiment outcome needs to be improved. The authors should clearly refer to centrosomal vs non-centrosomal nucleation centers and not just to "asters".

We agree with the reviewer that gamma-tubulin staining in nocodazole washout experiments in Fig. 5 does not distinguish between centrosomal and non-centrosomal nucleation centers. We repeated this experiment and will stain cells for centrin (centrosomal nucleation center), alpha-tubulin and DAPI. Once we analyze this new set of experiments, we will include data on centrosomal and non-centrosomal nucleation centers and discuss results accordingly.

8) The gtub staining in S5C seems abnormal. What is the explanation for this? I am not aware of such staining by normal IF.
For Fig. S5C, we performed Ultrastructure Expansion Microscopy to determine nano-scale organization of gamma-tubulin in control and CCDC66-depleted cells. Recent work by Luders lab used expansion microscopy and showed that gamma-TuRC localizes in the centriole lumen and wall in addition to its PCM and microtubule localization. (https://www.nature.com/articles/s41467-021-26252-5.pdf?origin=ppub). Analogously, our results indicate localization of gamma-tubulin to the inner centriole call, pericentriolar material and proximal spindle in the G2 cell. We included this data to show that abundance of gamma-tubulin at the PCM and spindle microtubules in mitotic cells is reduced upon CCDC66 depletion and to assess whether spatial organization of gamma-tubulin changes. We included the following sentence to detail these results and reference the paper:

“We also noted that organization of gamma-tubulin at the PCM was disrupted while its pool at the centriole wall and lumen remained intact, which was recently reported to be required for centriole integrity and cilium assembly (Fig. S5C) (Schweizer et al., 2021).”

9) Fig. 6CG,H: I am not sure what exactly was done here. The methods talk about flow chambers. Could the authors not simply incubate their proteins with taxol-stabilized microtubules and then spot/sediment/fix these on coverslips? Instead of microtubules the tubulin channel shows mostly dots/aggregates. These are more visible in the control. The authors should also show commassie gels of all purified proteins to estimate their purity. Only with relatively pure preparations in vitro experiments are interpretable, especially when proteins were obtained from a eukaryotic expression host. The technical aspects of these experiments seem of insufficient quality.
To test the direct microtubule bundling activity of CCDC66 and its C-terminal domain we used a previously well-described assay (Tao et al., 2016, King and Petry, 2020) using a flow chamber constructed with 22 × 22 mm coverslip taped to a glass slide with the double-stick tape. The motivation of using this method for the bundling assay was to avoid some microtubule bundles in the control that could appear as artifacts of the sedimentation process.

As suggested by the reviewer, we now performed these experiments by incubating our proteins with taxol-stabilized microtubules, fixed, and sedimented onto glass coverslips followed by staining with anti-tubulin antibody to visualize microtubules. This experiment is now added to Fig. 6G and it confirms previously observed microtubules bundles when incubated with His-MBP-mNeonGreen-CCDC66 but not with His-MBP-mNeonGreen control. Tubulin aggregates were observed in these assays due to our aged rhodamine-labeled tubulin, while there is less tubulin aggregation in the assays with only non-labeled tubulin (Cytoskeleton, Inc.).

As suggested by the reviewer, we added the Coomassie gel of His-MBP-mNeonGreen-CCDC66 and His-MBP-CCDC66 (570-948) purification to the previously shown His-MBP-mNeonGreen Coomassie gel in Fig. S6. Proteins His-MBP-CCDC66570-948, His-MBP-mNeonGreen and MBP were purified from bacterial Rosetta cells, and only tagged full-length CCDC66 protein was purified from the insect cells. Based on our one step-purification and Coomassie gel we agree that we cannot conclude the full-length CCDC66 protein preparation is of high purity. Therefore, we have further confirmed the presence of CCDC66 and identified most of the multiple ladder-like bands as degradation products by western blotting with antibodies against CCDC66, His tag and mNeonGreen tag. We endeavor to find conditions in which the protein will be more stable and more than 90% pure for our future single-molecule reconstitution assays, as well as SAX and CryoEM structural analyses. It is important to emphasize that although our full-length CCDC66 is obtained from a eukaryotic expression host and therefore other interacting proteins could have been pulled during purification, the C-terminal domain was purified from bacterial source and it also exhibit independent microtubule bundling activity as shown in Fig. 6H.

10) Fig. 7A: The localizations of CCDC66 proteins look very different from those in Fig. 1, despite the claim by the authors that they are similar. For example, there is almost no spindle microtubule staining. Again, there is no indication what the different expression levels are. Under the conditions shown, do the siRNA treatments actually deplete endogenous CDCC66 by western in all cases and do not affect

recombinant expression levels? The graphs at the bottom should indicate for each construct also depletion by siCCDC66.

We agree with the images in Fig. 7A we included in the manuscript do not represent spindle microtubule localization of mNG-CCDC66 in CCDC66-depleted cells, in particular for the first panel co-stained with gamma-tubulin. We went back through the data for quantified for rescue experiments and confirmed that mNG-CCDC66 localizes to spindle microtubules upon CCDC66 depletion. We now replaced these images with new ones that represent the spindle localization in Fig. 7A. Additionally, we confirmed that mNG-CCDC66, mNG-CCDC66 (570-948) and mNG-CCDC66-PACT is resistant to CCDC66 siRNA treatment in RPE1 cells using mNG antibody in immunoblotting experiments,. We will perform similar experiment in U2OS cells and include data confirming their siRNA-resistance.

Reviewer #2 (Comments to the Authors (Required)):

The manuscript by Batman et al. studies the role of CCDC66 in mitotic progression and cytokinesis by promoting centrosome maturation and microtubule bundling. Previous studies from the same laboratory determined the localisation of CCDC66 to the centrosomes, centriolar satellites, primary cilium and microtubules in different cell states (Conkar et al. 2017 and 2019). In addition, these studies and another's identified CCDC66 as a MAP and centrosome protein regulating cell division through interactions with regulators of cells division (Conkar et al. 2017, Gupta et al. 2015, Gheiratmand et al., 2019). Its depletion has shown spindle pole disorganisation in mitotic cells (Conkar et al. 2017, Sharp et al., 2011).

This study contributes to understanding the spatiotemporal regulation of critical events during Mitosis, such as the centrosome maturation during bipolar spindle assembly and the bundling activity of MT in the central spindle and midbody during cytokinesis. Specifically, the authors demonstrated that CCDC66 targets the centrosomal CDK5RAP2 proteins and Pericentrin. Notably, they show the CCD66 role in bundling MT as displayed by the disorganisation at the central spindle and midbody in CCDC66-depleted cells. Future studies will be required to understand how CCD66 regulates all these processes. Moreover, optogenetic and knocksideways approaches to inactivate and relocate CCDC66 protein, respectively, would help to discern the specific function involved at the different mitotic phases.

The results present in this work would be of interest to the general cell biological community in the field. Therefore, the interest of this study is important, and at this stage, the authors have supported by strong evidence their work and conclusions. Although, two minor points would support better some conclusions and findings discussed in the context of previous literature.

We thank the reviewer for the summary and constructive criticism of our manuscript. We are encouraged to see that the reviewer found our study of general interest and highlighted that our conclusions are supported by strong data. We previously applied FKBP-FRB-based redistribution approaches to manipulate centriolar satellite positioning, which allowed us to correlate their function with localization (Aydin et al. PLOS Biology 2021). As the reviewer suggested, we plan to apply knocksideways approach to examine CCDC66 function at different stages of cell division in future, following protocols used previously for other MAPs such as TACC3. Since development and application of this approach for this manuscript is not feasible, we included optogenetics and knocksideways approaches in the discussion as potential future avenues.

1-Previous studies in Conkar et al., 2017 the authors show the endogenous levels of CCDC66 in RPE1 cells by immunofluorescence. However, the authors in this work report that commercial and homemade antibodies did not detect endogenous CCDC66 levels by immunofluorescence. In this work, all the conclusions rely on the overexpression of CCDC66. The effects of overexpression need to be considered. The authors should add a western blot analysis to compare the expression level of these tagged proteins with the endogenous protein supporting Fig. S1A.

We agree with the reviewer's major concern, which was raised by all three referees. Below are new results that together address this concern and confirms localization of CCDC66 to spindle poles and multiple microtubule-based structures during cell division:

- For the first submission, we tested three antibodies raised against different CCDC66 antigens in localization experiments (Bethyl Laboratories A303-339A - antigen: 898-948 a.a; Sigma SAB1408484 - antigen: 1-831 a.a.; Home-made = antigen: 570-948 a.a.) Although we optimized antibody concentration in methanol, PFA or methanol/PFA-fixed U2OS cells, we had not observed consistent endogenous localization to mitotic structures. This is why we only included localization data of cells stably expressing CCDC66 fusion protein.

  For the revisions, we worked on further optimization of immunofluorescence protocols (fixation, blocking and antibody dilution steps) with the Bethyl antibody that we previously detected spindle pole and microtubule staining in RPE1 cells (Conkar et al. 2017). In U2OS cells fixed with methanol-acetone, Bethyl antibody generated consistent results to demonstrate co-localization of endogenous CCDC66 to the spindle poles and microtubule-based structures of cell division (bipolar spindle, central spindle, midbody) despite high cytoplasmic and punctate background (Fig. 1A and S1A). Using U2OS cells depleted for CCDC66, we also confirmed the specificity of the antibody in detecting the mitotic and cytokinetic structures (Fig. S3B)

- We examined localization of GFP-CCDC66 using a previously characterized RPE1::GFP-CCDC66 cell line that expresses near-endogenous levels of GFP-CCDC66 (Conkar et al. JCS 2017). Like mNG-CCDC66 localization in stable lines, GFP-CCDC66 also localizes to the bipolar spindle in metaphase and midbody in cytokinesis. We note that we used mNeonGreen-CCDC66 in our manuscript as its brightness and photostability is advantageous for live imaging experiments.

- We used lentivirus infection followed by puromycin selection to generate U2OS::mNG-CCDC66 and RPE1::mNG-CCDC66 stable lines. We prepared lentivirus using an expression vector that has EF1 promoter (pCDH-EF1-MCS-T2A-Puro). Using CCDC66 antibody, we examined expression levels of mNeonGreen-CCDC66 in U2OS stable lines. Despite the punctate background, the western blot data demonstrates that mNeonGreen-CCDC66 is expressed at near-endogenous levels in the U2OS stable line (Fig. S1D) . We will also quantify mNG-CCDC66 expression levels relative to endogenous protein in RPE1::mNG-CCDC66 cell line.

Taken together, these results show that CCDC66 localizes to spindle poles and multiple microtubule-based structures during cell division and confirms that mNeonGreen-CCDC66 localizes similar to the endogenous protein.

2-The authors show that mNG-CCDC66(570-948) localises to the spindle poles, bipolar spindle, central spindle and midbody upon transient expression in mitotic cells demonstrating, microtubules association and microtubule bundling. Previous literature, Conkar et al., 2017, showed that the N-terminal region, 564 amino acids that contain the CCDC66 domain, was able to localise and interact with microtubules. Could the authors confirm if this N-Terminal fragment recapitulates its localisation interacting with microtubules during Mitosis? This result would not only be very reassuring for the general significance of the study but would also allow the authors to confirm that r the N-terminus does not induce the formation of microtubules bundles and only decorates the microtubule network in the cells.
Thhe reviewer raises an important question regarding how different CCDC66 domains contribute to its mitotic function via binding to microtubules. As the reviewer noted, we previously showed that N-terminal CCDC66 fragment binds and localizes to microtubules in cells. Since we have not performed in vitro assays with purified proteins, we do not know whether this fragment binds to microtubules directly and if so, whether it affects microtubule organization.

To address the reviewer's question on whether CCDC66 (1-570) localizes to spindle microtubules, we transfected U2OS cells with mNG-CCDC66 (1-570) and examined its localization in cells at different stages of cell division. As shown below, we observed localization of this truncation mutant to the spindle poles and bipolar spindle in metaphase and centrosomes and midbody in cytokinesis in a fraction of transfected cells. To determine whether it exhibits a similar localization pattern in stable lines and whether this localization persists upon CCDC66 depletion, we will try to generate U2OS cells stably-expressing

this fragment. We would like to highlight the technical issues we experienced previously in generating cell lines that stably express some of the CCDC66 fragments, likely due to the toxic effect of constitutive expression of these fragments.

To address the reviewer's comment on whether the microtubule bundling activity of the mNG-CCDC66 (570-948) is sufficient to rescue microtubule bundling-associated mitotic phenotypes, we will perform phenotypic rescue experiments for k-fiber integrity using mNG-CCDC66 and mNG-CCDC66 (570-948)-expressing cell lines (Fig. 4D). If CCDC66 (570-948) rescues this phenotype, we can propose that its microtubule-bundling activity is sufficient for CCDC66 functions at the k-fibers. In case of no rescue, we will propose that N-terminal domain is also required for this function.

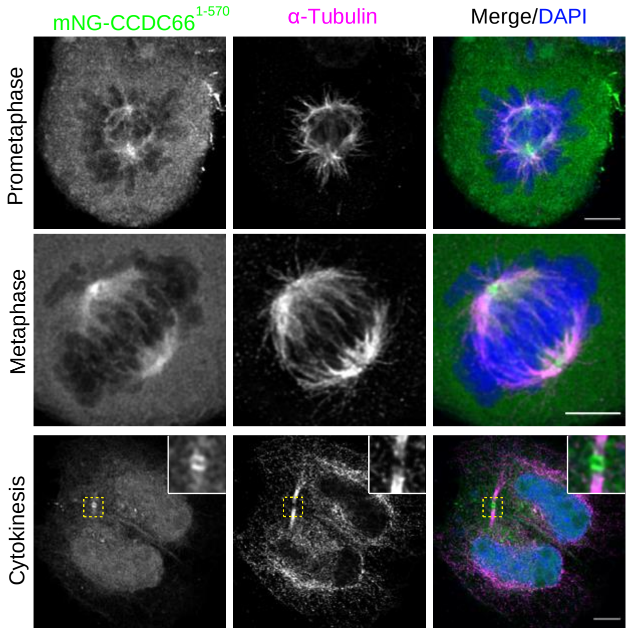

Reviewer #3 (Comments to the Authors (Required)):

The manuscript by Batman et al characterizes the role of the protein CCDC66 in mitosis.
The authors detect this protein both at the pericentriolar material and at the midbody, and identify several proteins of the centrosome and of the midbody as potential interactors by co-immunoprecipitation. Depletion of the protein leads to mitotic defects with abnormal spindles and reduced kinetochore fibres and reduced astral microtubules, as well as problems in cytokinesis, such as failure of abscission or reversal of the cleavage furrow. The data suggest a role of CCDC66 in the centrosomal recruitment of nucleation factors, as well as a direct role in the bundling of microtubules, such as midbody-microtubules during cytokinesis.

Generally, I think this is a manuscript that should be of interest to a wide readership in cell biology. The manuscript could be further improved; I have the following comments:
We thank the reviewer for the summary and constructive criticism of our manuscript. We are encouraged to see that the reviewer found our study of general interest.

1) CCDC66-interactors are identified by co-immunoprecipitation of co-transfected, overexpressed proteins. Ideally, experiments on co-precipitation of endogenous proteins would be the best experiments, since co-precipitation of pairwise overexpressed proteins may risk to produce artifacts. I am aware that low abundance and low solubility of endogenous proteins may represent unsurmountable obstacles to

such an approach. Nevertheless, the authors could add a sentence in their manuscript, discussing the risks and the limits of their experimental strategy.
We agree with the reviewer that pulldowns with endogenous proteins will be the ideal experiment. We also thank the reviewer for acknowledging the challenges in accessing the insoluble and low abundance centrosome interactions using traditional pulldown experiments. In addition to performing pulldowns in cells overexpressing fusion proteins, we and others have extensively used proximity-labeling approaches to overcome these challenges. We note that the BioID-based proximity interactome of CCDC66 identified CDK5RAP2, CEP192 and CEP152 as proximity interactors, further supporting the interactions between CCDC66 and PCM proteins. We now included the following sentence in discussion:

"Given the power of proximity-mapping approaches in accessing insoluble and low abundance interactions, we next asked whether the interactions we identified using co-immunoprecipitation experiments are reflected at the proximity level (Conkar et al., 2017; Gheiratmand et al., 2019; Gupta et al., 2015). The CCDC66 proximity map included CDK5RAP2, CEP152 and CEP192, but not CEP55 and PRC1, which might be due to the lower abundance of cytokinetic interactions in datasets generated from asynchronous cultures."

2) On the myc blot in Figure 2E, the bands of mycBirA should be shown in one image (instead of two separated ones), to prove that mycBirA is a reliable negative control.
When we ran this control blot, the loading order was different than the presented one and thus, we cropped the parts of the blot that includes initial sample and eluate. We now included a new blot to indicate that myc-BirA* does not co-pellet with FLAG-miniTurbo-CCDC66 (Fig. 2E). As another specificity control, we now included co-IP results from cells co-expressing FLAG-miniTurbo control with myc-BirA* fusions of CDK5RAP2, CEP152 and CEP192 (Fig. 2E)

3) Figure 4: the differences in spindle angle and spindle length after CCDC66-depletion are not very big. It is puzzling that astral microtubules are shorter while the pole-to-pole distances of the spindles are shorter, as I would expect the opposite (longer astral microtubules). In this context, I am wondering whether the increased tilt angle of the spindles has been taken into account for the calculation of the distances between the spindle poles? A tilted spindle appears shorter when viewed from the top, even if the pole-to-pole distance is the same! Can the authors re-visit their raw data, and eventually adjust the calculations?
We thank the reviewer for the great suggestion. We agree that tilted spindles could appear shorter when viewed from the top. As suggested by the reviewer, we will reanalyze our raw images to take into account the impact of the tilt angle of the spindles on spindle length for Fig. 4. To ensure consistency throughout the manuscript, we will also reanalyze the raw images of phenotypic rescue experiments we performed for spindle angle (Fig. 7). Additionally, we will also quantify spindle angle during our reanalysis. $\alpha=180*\tan-1(h/L)/\pi$ is the formula we used to calculate spindle angle where h represents the stack difference between two spindle poles and L represents the distance between spindle poles when projected to the same stack. Therefore, this formula will allow us to analyze both spindle length by taking into account the tilt angle of spindles.

4) Figure 5D: what are the criteria for so-called "prometaphase-like spindles"? Shouldn't one use a more precise and more descriptive terminology?
We agree with the reviewer and categorized the spindles as "prometaphase", "monopolar", "bipolar" and "disorganized" in the revised Fig. 5D.

5) Figure 6E: shouldn't the labeling of the figure for alpha-tubulin be in pink? Green is PRC1?
We corrected the mis-labeling in Fig. 6E.

6) It would be most interesting to see rescue experiments for the cytokinesis phenotype (using the three CCDC66 constructs as shown in Figure 7).
As suggested by the reviewer, we have started performing experiments to determine which CCDC66 fusion protein rescues the cytokinesis defect. We will quantify the percentage of multinucleated cells and include in the revised manuscript.
