# Supplementary material for: The ciliopathy protein CCDC66 controls mitotic progression and cytokinesis by promoting microtubule nucleation and organization
Source: PLoS Biol. 2022 Jul 18;20(7):e3001708. doi: 10.1371/journal.pbio.3001708 (PMC9333452; doi:10.1371/journal.pbio.3001708)
Supplement: Attachment — Submitted filename: PointByPointResponse_final.pdf [file pbio.3001708.s020.pdf]

Reviewer #1:

In this manuscript, the authors examine the contribution of the ciliopathy protein CCDC66 to mitosis and cytokinesis. The authors show that the protein localizes to mitotic microtubules in dividing cells including spindle kinetochore fibers, PCM and midbody using both immunofluorescence and expression of the tagged exogenous protein. CCDC66 contribution to the localization of other PCM components, binding partners and microtubule crosslinking activity of CCD66 are determined. For the most part the work provides new information that will be of interest to cell biologists.

We thank the reviewer for the constructive criticism of our manuscript. We are encouraged to see that the reviewer acknowledged the novelty of our results and found our study of interest to cell biologists.

1. To study CCD66 in mitotic cells, the authors deplete the protein with siRNA, which is verified with an immunoblot and by immunofluorescence. To more clearly depict the mitotic fate of the cells that were depleted of CCDC66, it would be good to show each cell as a bar representing the duration of mitosis and indicating the fate. An example of this type of representation see Wong, et al Science 2015, Figure 4.

We re-analyzed live imaging data from control and CCDC66-depleted cells to determine fates for individual cells. Specifically, we followed the dividing cells during 12 h of imaging and determined their fate as well as mitotic time for the cells that successfully complete mitosis. Given the duration of live imaging we performed (12 h), we only included analysis of the mother cell fate, but not that of the daughter cells as previously described in Wong et al. Science 2015 (PMID: 25931445) and Uetake Y et al. Curr Biol 2010 (PMID: 20832310) papers. The representative graphs for cell fate analysis are presented in Fig. S3C. In these graphs, each vertical bar in these graphs represents an individual cell. Bar height is the time spent in mitosis after nuclear envelope breakdown (NEBD), and color indicates successful division (gray), arrest (pink) or apoptosis (cyan). Comparative analysis of these graphs further supports the defects in mitotic progression associated with CCDC66 loss (Fig. 3A-D). The fraction of cells that underwent apoptosis or arrest as well as the mitotic time were higher in CCDC66-depleted cells as compared to control cells.

2. The defects in astral microtubules and spindle positioning and the localization of CCDC66 at the centrosome are consistent with a role for CCDC66 in microtubule nucleation. This is not a surprising result for a protein at the PCM that binds to gamma tubulin. The images of astral microtubules in the stained cells are not particularly convincing, and better images would be nice to see.

For endogenous CCDC66 staining in human cell lines, we tested three antibodies raised against different human CCDC66 antigens in localization experiments (Bethyl Laboratories A303-339A - antigen: 898-948 a.a; Sigma SAB1408484 - antigen: 1-831 a.a.; Custom = antigen: 570-948 a.a.) After extensive optimization of staining protocols, we showed that the Bethyl antibody revealed specific localization of endogenous CCDC66 in U2OS cells to the spindle poles and microtubule-based structures of cell division (bipolar spindle, central spindle, midbody) (Fig. 1A and S1A). However, the antibody had high cytoplasmic, punctate background. Using U2OS cells depleted for CCDC66, we confirmed the specificity of the antibody in detecting the mitotic and cytokinetic structures (Fig. S3B).
Likely due to the background associated with the antibody staining and lower abundance of CCDC66 at the astral microtubules compared to the bundled k-fibers, we detected very weak signal at the astral microtubules in human cells (Fig. S1A). The latter possibility is supported by prominent localization of mNG-CCDC66 to the astral microtubules in RPE1 stable cells (Fig. S1E). We now included a new image in Fig. S1A that is representative of this conclusion. Moreover, we included the following sentences to clarify these points in pg. 9:

“Although CCDC66 localization to the astral MTs and midzone was apparent in cells stably expressing mNG-CCDC66, it was very weak in cells stained for endogenous CCDC66 (Fig. 1A and S1A). This might be due to the high cytoplasmic and punctate background associated with CCDC66 antibody staining and/or the relatively lower abundance of CCDC66 at the astral microtubules and spindle midzone.”

3. Given the ability of CCDC66 to bind and bundle microtubules and its co-localization with PRC1 in the midbody, the authors hypothesize that CCDC66 contributes to cytokinesis. Consistent with this, the authors state that a large percentage of cells depleted for CCDC66 display asymmetric furrows indicating a defect in cytokinesis. However, I had some questions about other aspects of cytokinesis.

- Midzone localization in anaphase cells is weak - it is present in the expressing cells but not apparent in the stained cells. This should be noted or clarified.

- As compared to astral microtubules (point#2), spindle midzone staining in anaphase was more apparent when cells were stained with the CCDC66 antibody (Fig. 1A and S1A). However, the antibody staining results in high cytoplasmic and punctate background and thus the signal/noise ratio at the central spindle is low. This might also be due to the lower abundance of CCDC66 at the midzone relative to its other MT-associated pools. We now included a new image in Fig. 1A and S1A that is representative of the central spindle staining of endogenous CCDC66. Moreover, we included the following sentences to clarify these points in pg. 9:

"Although CCDC66 localization to the astral MTs and midzone was apparent in cells stably expressing mNG-CCDC66, it was very weak in cells stained for endogenous CCDC66 (Fig. 1A and S1A). This might be due to the high cytoplasmic and punctate background associated with CCDC66 antibody staining and/or the relatively lower abundance of CCDC66 at the astral microtubules and spindle midzone."

- During the revision process, we generated a custom antibody against full-length mouse CCDC66, which works very well for endogenous CCDC66 staining in mouse cell lines but not human cell lines. As presented below, the mouse CCDC66 antibody stains the bipolar spindle, the midzone in anaphase cells, the central spindle and midbody at other stages of mitosis in mouse kidney epithelial (IMCD3) cells. We confirmed the specificity of the antibody in mouse cell lines depleted for CCDC66 using shRNA. Since our manuscript currently does not have data generated using IMCD3 cells, we did not include the following data to the revised manuscript. If the reviewer recommends that these staining data will strengthen our conclusions, we will include the IMCD3 staining data in the supplemental Fig. 1.

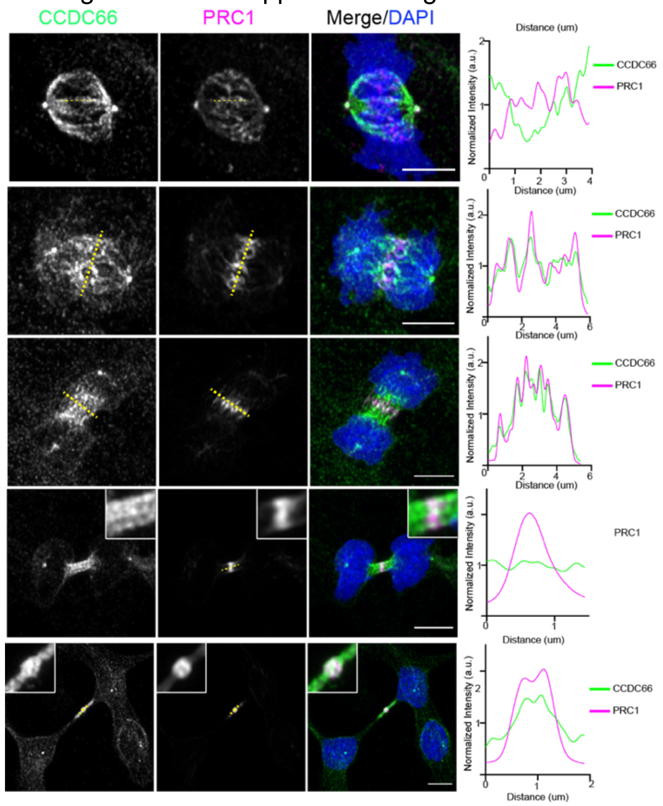

- CCDC66 interacts with PRC1 and co-localizes with PRC1 at the midbody in late telophase/cytokinesis. Do CCDC66 and PRC1 co-localize at the anaphase midzone? Given the staining in Fig 1, I think not. Please clarify.

In the revised manuscript, we included a representative image of RPE1::mNG-CCDC66 cells stained for PRC 1 during telophase and cytokinesis (Fig. S2A). The plot profile analysis of their relative localization indicates that mNG-CCDC66 and PRC1 co-localize at the spindle midzone.

The Bethyl CCDC66 and PRC1 antibodies that work in immunofluorescence experiments in human cells were both generated in rabbit. Therefore, we were not able to perform co-localization experiments for endogenous PRC1 and CCDC66 in human cells. As an alternave, we co-stained IMCD3 cells for endogenous CCDC66 (rat antibody) and PRC1 (rabbit antibody). As shown above in the IMCD3 staining data, endogenous PRC1 and

CCDC66 co-localize in the spindle midzone in anaphase cells. This result is in agreement with the localization data we report for mNG-CCDC66 (Fig. S2A)

- The additional "spots" of fluorescence of CCDC66 in the midzone (distal to the co-localization with PRC) should be quantified to be sure these are not aggregates of the expressed protein. What % of cells show these? Are they observed in fixed and stained cells or only the expressing cells?

As the reviewer highlighted, we noted the accumulation of mNG-CCDC66 outside the PRC1 ring during cytokinesis (Fig. 2). This staining profile was prominent in all cells at later stages of cytokinesis, which is nicely represented in early/late cytokinesis staining in Fig. S1E, the still images from Movie 1 in Fig. S1F as well as the figure presented below regarding localization of mNG-CCDC66 relative to PRC1. To define this structure, we performed co-localization experiments of mNG-CCDC66 with known midbody markers (Fig. 2D). However, we did not find co-localization with the markers we tested neither did we find a report of a similar structure in literature.

Therefore, these puncta is most likely just accumulation of mNG-CCDC66 at the microtubule structure of intercellular bridges (outside of the midbody) and a consequence of CCDC66 ectopic expression. It is not prominent with the antibody staining in IMCD3 and U2OS cells. We clarified this point in the manuscript by including the following sentence in pg. 11:

“Of note, we observed accumulation of CCDC66 outside the midbody in cells at later stages of cytokinesis, which is also evident in the dynamic behavior of mNG-CCDC66 during cell division (Fig. 2D, Movie 1, Movie S1). These CCDC66-positive structures did not co-localize with the known midbody markers and were not detected in cells stained for endogenous CCDC66 (Fig. 1A).

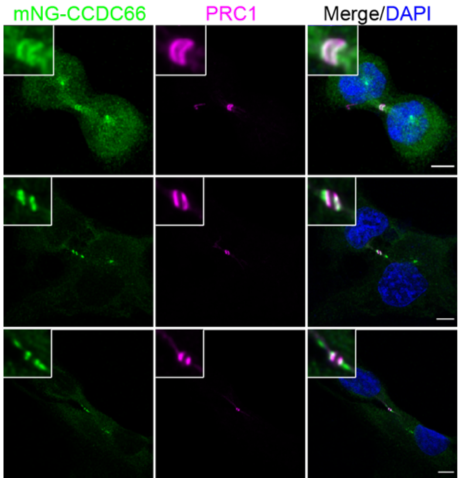

- The data in Fig S3 show midzone microtubules, in control and CCDC66 depleted cells, but defects but not quantified. The examples suggest subtle differences given the cell-to-cell variation in midzone microtubule organization. This needs to be quantified or removed.

As suggested by the reviewer, we quantitatively analyzed the central spindle defects associated with CCDC66 depletion using three different analyses approaches.

1- We quantified the percentage of control and CCDC66-depleted cells that exhibit disrupted organization of central spindle MTs. We specifically quantified cells in anaphase by using DNA staining as a marker for determining the cell cycle stage (shown in the inset in Fig. 5A). 57.04% CCDC66-depleted cells had disorganized arrays of central spindle MTs and failed to form an ordered centered array, indicating defects in MT bundling in anaphase (Fig. 5C). In contrast, only 20.32% of control cells had this defect (Fig. 5C).
2- We quantified the microtubule intensity at the central spindle in anaphase cells and found that it was significantly reduced upon CCDC66 depletion (Fig. 5B). This results further supports roles of CCDC66 in non-centrosomal MT nucleation.
3- Given the co-localization and interaction of CCDC66 with PRC1, we examined whether CCDC66 is required for PRC1 localization and recruitment to the central spindle. The spatial distribution of PRC1

levels at the central spindle were disrupted in CCDC66-depleted cells relative to control cells (Fig. 5H). Specifically, PRC1 spread out along microtubules rather than exhibit a focused localization at the central spindle.

Collectively, these new results demonstrate that CCDC66 is required for central spindle assembly and organization. We included these results and its discussion as a new figure in Fig. 5 and S5.

- The cytokinesis defects could result from defects in spindle formation that are manifest later on at cytokinesis. Another possibility is that CCDC66 contributes not to midzone organization in anaphase, but in the abscission process.

We agree with the reviewer that the cytokinesis defect associated with CCDC66 depletion can be explained by multiple mechanisms including 1) defective midzone organization in anaphase, 2) defective spindle formation manifested later during cytokinesis (if astral microtubules are not bound to cortex, then signal for midzone formation is missing), 3) defective abscission process (mislocalized and/or defective recruitment of abscission factors). While our results provide support for the first two mechanisms, we cannot eliminate the third one as we did not dissect the molecular and dynamic defects in cytokinesis upon CCDC66 loss. In this manuscript, we focused on dissecting the mitotic functions and mechanism of action of CCDC66. In future studies, we plan to focus on uncovering cytokinetic functions and mechanisms of CCDC66. We now discussed these possibilities in pg. 23 and 24 in the discussion section.

4. The authors use microtubule regrowth assays to ask if the loss of CCDC66 impacts aster size and spindle formation following washout. They use STLC treated cells presumably so that a mono-aster is measured. But in the figure, the CCDC66 siRNA cell shown has two spots of gamma tubulin - it did not form a monopolar spindle? Is this a common phenotype? is the quantification of microtubule length for single asters (presumably the centrosomes separate on washout)?

As the reviewer noted, we treated cells with STLC to induce formation of monopolar spindles and synchronization, which was followed by nocodazole treatment and washout. Majority of the cells had monopolar spindles after STLC treatment, and we replaced the representative image for control cells to reflect this phenotype at t=0 (Fig. 6A). We note that centrosomes were proximate but still separate in these cells, likely due to nocodazole treatment of STLC-treated cells.

To distinguish between centrosomal and non-centrosomal nucleation sites after nocodazole washout, we stained cells with centrin (centrosomal) instead of gamma-tubulin in new sets of experiments. We performed two different analyses in these cells. First, to assess their microtubule nucleation capacity, we quantified the microtubule aster size by drawing a freehand shape around the edges of the microtubules at the aster and quantified the area. The microtubule aster size at the centrosomal and non-centrosomal nucleation centers were reduced upon CCDC66 depletion at 3, 5 and 8 min after nocodazole washout (Fig. 6B). Second, we quantified the number of non-centrosomal nucleation sites, which were higher in CCDC66-depleted cells as compared to control cells (Fig. S6A). This suggests that CCDC66 loss might activate non-centrosomal MT nucleation.

As for the quantification of the microtubule aster size nucleated by centrosomal and non-centrosomal nucleation sites, we used the freehand tool to draw the area around the microtubule ends at different time points and plotted it in Fig. 6B. We revised the related methods section to detail quantification of microtubule aster size.

5. The authors use knockdown and rescue to determine if the full-length protein, the microtubule binding C-terminal fragment and/or CCDC66 that is targeted to the centrosome with a PACT domain can rescue three aspects of the depletion phenotype: gamma tubulin at poles, spindle microtubule intensity and spindle length. The conclusion of the rescue experiments is that all three constructs can rescue these phenotypes, suggesting that CCDC66 at the centrosome is sufficient. In the discussion the authors state that "full length and C-terminal domain rescued these phenotypes to a greater extent than the CCDC66PACT" - although that is not clear from figure 7. Spindle length looks very similar in all cases and the PACT domain construct results more spindle tubulin.

As the reviewer noted, the spindle length phenotype we reported in the original manuscript was significant but was minor (0.935-fold decrease). Based on Reviewer 2's feedback (point 1), we reanalyzed the spindle length phenotypes by taking into account the impact of the tilt angle of the spindles on spindle length. The results from this analysis showed that CCDC66 depletion does not result in a significant spindle length phenotype (Fig. 4A).

In the light of these results, we changed our conclusions in the revised manuscript and removed the rescue experiments for the spindle length phenotype.

For the revised manuscript, we performed four new phenotypic experiments. Specifically, we examined whether expression of mNG fusions of CCDC66, CCDC66 (570-928) and CCDC66-PACT rescues the defects in k-fiber stability, central spindle MT intensity and organization, spindle positioning and cytokinesis. The result of the rescue experiments is detailed below. As for cytokinesis defect, we found that CCDC66 and CCDC66 (570-948) restored this defect. However, CCDC-PACT did not restore it (Fig. 7D, F). These results show that MT-binding activity of CCDC66, but not its centrosome-mediated MT nucleation activity, is required for its functions during cytokinesis.

- The three phenotypes measured all relate to the microtubule nucleating activity; are cytokinetic defects seen with the PACT containing construct? Is microtubule binding contributing to microtubule nucleation? I suppose resolving these issues will require use of constructs that contribute to a single function - nucleation or microtubule binding -- and are thus likely outside the scope of the present study. Nevertheless, the authors should be as clear as possible about the results of these experiments, which are impacted by possible overexpression of the rescue construct and additional centrosome binding in the case of the PACT construct.

- Although we generated various CCDC66 mutants in the lab (Conkar et al. JCS 2017), we were not able to identify the minimal regions required for microtubule nucleation and bundling as well as centrosome and microtubule localization. It is possible that these activities are mediated by overlapping regions of CCDC66 and as such, identification of a domain that contribute to a single function is not possible. Therefore, we cannot perform these experiments suggested by the reviewer. We thank the reviewer for acknowledging that these studies will be outside the scope of the present study. In the revised manuscript, we included the following sentence in pg. 26 to discuss the drawbacks of the rescue experiments associated with overexpression of the rescue construct and the additional centrosomal binding sites created by the PACT fusion.

"*Of note, the strong centrosome affinity of the PACT domain increased CCDC66 levels at the centrosome and created more binding sites for its interactors such as gamma-tubulin, which might have compensated for lack of MT association for a subset of CCDC66 functions."*

- For the revised manuscript, we performed 5 new rescue experiments for K-fiber integrity, central spindle intensity and organization, cytokinesis and spindle positioning defects to distinguish between centrosomal and non-centrosomal microtubule nucleation as well as MT organization activities of CCDC66. We performed these experiments in cells stably expressing mNG, mNG-CCDC66, mNG-CCDC66 (570-948) and mNG-CCDC66-PACT and came to conclusion that indeed microtubule binding of CCDC66 contributes to non-centrosomal MT nucleation. Below is the summary of these experiments:

A) In Fig. 7E, we plotted **central spindle microtubule intensity** of the stable cell lines transfected with control or CCDC66 siRNA. Expression of mNG-CCDC66 and mNG-CCDC66 (570-948) restored the decrease in central spindle microtubule intensity associated with CCDC66 depletion. The centrosome-restricted mNG-CCDC66-PACT did not restore this phenotype. This experiment together with nocodazole washout experiment in new Figure 6B, where we identified defects in acentrosomal microtubule asters, provide strong evidence that CCDC66 is involved in non-centrosomal microtubule nucleation and through its microtubule binding activity. We further quantified rescues of **aberrant central spindle formation** in Figure S7F and again mNG-CCDC66 and mNG-CCDC66 (570-948) restored this defect while mNG-CCDC66-PACT did not, further supporting the role of CCDC66 microtubule bundling activity in organization of central spindle in anaphase.

B) In Fig. 7F, we plotted percentage of **binucleated cells** of the stable cell lines transfected with control or CCDC66 siRNA. Expression of mNG-CCDC66 fully and mNG-CCDC66 (570-948) partially restored the cytokinesis defect in CCDC66-depleted cells. However, mNG-CCDC66-PACT did not restore this phenotype, indicating that microtubule-binding and bundling activity of CCDC66 is required for its functions during cytokinesis. The partial rescue by mNG-CCDC66 (570-948) shows that its N-terminus also contributes to CCDC66 functions during cytokinesis. N-terminus might contribute to cytokinesis by binding to and regulating microtubules and/or by recruitment of cytokinetic factors to the spindle midzone. Taken together, these results together support that the microtubule bundling activity of CCDC66 is required for cytokinesis.

C) In Figure S7E, we plotted microtubule intensity at the **K-fibers** in cold-treated cells. Expression of mNG-CCDC66 and mNG-CCDC66 (570-948) both rescued these phenotypes. However, mNG-CCDC66-PACT did not. These results show that MT binding activity of CCDC66 is important for their stability and organization.

D) In Fig. S7C, we plotted the **spindle angle** of the stable cell lines transfected with control or CCDC66 siRNA. Expression of mNG-CCDC66, mNG-CCDC66 (570-948) and mNG-CCDC66-PACT all rescued the spindle positioning defects associated with CCDC66 depletion. The rescue by mNG-CCDC66-PACT can be explained by the increased microtubule nucleation from the spindle poles in part due to higher levels of gamma-tubulin. As for mNG-CCDC66 (570-948), it might contribute to spindle positioning via stabilizing microtubules. These results show that CCDC66 functions during spindle positioning by regulating centrosomal microtubule nucleation as well as stability/organization of astral microtubules

We now included the results from the new phenotypic rescue experiments in Fig. 7 and Fig. S7 and discussed the results in the discussion section in terms of their implications in our understanding of relative contributions of CCDC66 activities and cellular pools to its mitotic and cytokinetic functions.

- Are the differences in gamma level, spindle length and microtubule intensity for the full-length and PACT constructs significantly different (from each other) not just as compared to the depleted (and not rescued) cells?

We performed statistical analysis for gamma tubulin levels and spindle microtubule intensity between full-length and PACT constructs. Our results showed that they are significantly different from each other. We included results of this analysis in the graph in Fig. 7B and 7C.

Minor

1- I do not see CCD66 on astral microtubules in figure S1E; please provide single color image of the CCD66 fluorescence.

We included single color images for CCDC66 staining in Fig. S1E.

2- The overexpression of CCD66 clearly results in artefacts as characterized in Figure 1D; thus, it is important that the level of expression be shown for rescue experiments and that the authors draw conclusions based on endogenous protein localizations.

We included two blots confirming the expression of the mNG fusions of CCDC66, CCDC66 (570-948) and CCDC66-PACT in cells transfected with CCDC66 siRNA. The blots in Fig. S7B together with the immunofluorescence data (Fig. 7) confirm that the fusion proteins are expressed at the right size and are resistant to CCDC66 siRNA. Although the western blot data indicates overexpression of the fusion proteins relative to endogenous protein, this does not reflect the expression level of the fusions in individual cells. The stable lines we used for rescue experiments were not 100% homogenous in terms of the expression levels of the fusion proteins. For quantification of the rescue experiments, we included the cells in which the fusion proteins were expressed at lower levels and localized properly to centrosomes and/or microtubules (representative images in Fig. 7). We revised the related methods section to detail how we performed quantification. It is of importance to note that stable mNG-CCDC66 and CCDC66 (570-948) cell lines are exhibiting lower levels of CCDC66 on MTs after longer time in cell culture due to the toxic effect of bundling and over-stabilization of MTs on cell division.

3- Page 9 the word "not" is missing when the interaction with mycBirA is described.

We included "not" in the related sentence in pg. 12.

4- In Movie 4 there are two mitotic cells; the one to the left looks like it undergoes apoptosis and the one on the left appears to undergo unequal division. In movie 5 there are also two mitotic cells; presumably the cell on the right is not depleted? Please add additional information.

We included the following sentence in the movie legend and put an asterisk to the cell exhibiting the indicated defect in the movie.

“The cell marked by the asterisk exhibits the apoptosis phenotype”

5- In figure 4 some panels have dots of two colors and others three; the colors represent different experimental trials—please clarify in the legend which experiments were repeated three (or two) times.

We specified the number of replicates analyzed for different phenotypes in the figure legends throughout the manuscript, including Fig. 4.

Reviewer #2 (previous LSA reviewer):

The revised version of the manuscript by Batman et al contains improvements that justify now consideration for a journal of higher visibility (such as PLOS Biology). In particular, the new data on endogenous CCDC66 represent a significant step forward.

We thank the reviewer for the summary and constructive criticism of our manuscript. We are encouraged to see that the reviewer found the initial revisions a significant step forward.

However, several of my earlier suggestions were apparently not followed:

The draft rebuttal letter we sent to PLOS Biology included data addressing several major concerns (i.e endogenous CCDC66 localization) as well as our strategy for how we will address the rest of the comments. Since we only had a month between receiving the reviewer comments from LSA and submission to PLOS Biology for initial evaluation, we did not have enough time to finish all the rebuttal experiments. We hope that this explanation clarifies the reviewer’s concern regarding the lack of experimental data in the first rebuttal letter. We now have experimentally addressed the reviewer’s concerns as detailed below.

1- I had criticized that spindle length in projected images appears shorter if spindles are tilted, and suggested that the length should be re-calculated from 3D data sets. Despite an appeasing response in which the authors promised that they will re-calculate ("As suggested by the reviewer, we will reanalyze our raw images to take into account the impact of the tilt angle of the spindles on spindle length for Fig. 4"), the data in the new Figure 4 are identical to the old version of the manuscript.

We thank the reviewer for the great suggestion. As suggested by the reviewer, we reanalyzed our raw images to take into account the impact of the tilt angle of the spindles on spindle length for Fig. 4A. $\alpha=180*\tan-1(h/L)/\pi$ is the formula we used to calculate spindle angle where h represents the stack difference between two spindle poles and L represents the distance between spindle poles when projected to the same stack. Therefore, this formula allowed us to analyze spindle length by taking into account the tilt angle of spindles. We now included the revised methods for spindle length quantification in the methods section.

The original analysis we performed for spindle length revealed a 0.935-fold decrease in spindle length. However, the results from the reanalysis showed that CCDC66 depletion does not result in a significant spindle length phenotype. In the light of these results, we changed our conclusions in the revised manuscript and removed the rescue experiments for the spindle length phenotype.

2- Rescue experiments for the cytokinesis phenotype were suggested. The authors state "As suggested by the reviewer, we have started performing experiments…. We will quantify … in the revised manuscript." However, the revised manuscript does not contain any such experiments.

As suggested by the reviewer, we investigated whether and if so to what extent stable expression of mNG fusions of CCDC66, CCDC66-Cterm and CCDC66-PACT rescues the cytokinesis defect. While mNG-CCDC66 fully restored the defect, mNG-CCDC66-Cterm (570-948) partially restored it. Strikingly, mNG-PACT-CCDC66 did not restore the cytokinesis defect. The results of these experiments allowed us to conclude that 1) cytokinesis defect is specific to loss of CCDC66, 2) evaluate the contribution of microtubule and centrosome association on cytokinetic progression as regulated by CCDC66.

Stabilization and bundling of MTs is essential for the assembly and maintenance of the MT arrays of cell division such as the midbody and thereby is required for cytokinetic progression. Given that microtubule-binding

CCDC66 (570-948) fusion but not centrosome-restricted CCDC66-PACT rescues the cytokinesis defects, our results indicate that CCDC66 operates during cytokinesis via its microtubule-bundling activity.

In addition to the rescue of the cytokinesis defect, we also performed rescue experiments for defects in k-fiber stability, spindle positioning and central spindle tubulin levels and organization. Collectively, the results from these experiments provided critical insight into the mechanisms by which CCDC66 regulates mitosis and cytokinesis. The results from these experiments and our interpretation is detailed below in response to the reviewer 3's major concern.

3- I had criticized that in Fig. 5D, establishing a category of "prometaphase-like spindles" is not precise enough to describe the phenotype. Now, the authors labelled the same figure with the description "prometaphase spindles". This terminology is equally unsatisfactory, since spindle formation can be a long procedure even during a regular prometaphase, starting from monopolar arrays (if centrosomes aren't already separated at the time of nuclear envelope breakdown), transiting to somewhat "disorganized" spindles, and finishing with bipolar spindles. The authors must tell in descriptive terms what they see, for example "spindles with unfocused poles", or "spindles with low MT density", or other. The present classification makes little sense, since a typical "prometaphase spindle" does not exist.

We agree with the reviewer. In the revised Fig. 6D, we categorized the spindles as "bipolar spindles with low MT density", "bipolar spindle", "monopolar spindle" and "disorganized spindle". Disorganized spindle refers to the spindles where spindle poles and microtubules are disorganized.

Overall, I am not satisfied with the revisions, and I suggest that additional improvements should be made.

In the revised manuscript, we experimentally addressed the concerns raised by the reviewer. The new set of experiments we performed based on the comments from 4 reviewers strengthened our conclusions. We hope that the reviewer agrees.

Reviewer #3 (previous LSA reviewer):

Thank you to the authors to review the manuscript. They have addressed a critical number of experiments to reassure some of the reviewers' concerns;

- The authors have reassured the critical point of this work regarding the overexpression of CCD66. They show the endogenous level of CCDC66 by immunofluorescence and western blot in U2OS. Also, they demonstrate the localization to the endogenous levels at the spindle poles and microtubules. Moreover, they have examined the protein levels of endogenous versus exogenous in the U2OS stable cell line.
- The authors have included some essential controls in the manuscript, replaced some representative images, and edited the manuscript to clarify some conclusions.

1) The authors mention in the rebuttal letter the importance to clarify whether the microtubule bundling activity of the mNG-CCDC66 (570-948) is sufficient to rescue microtubule bundling associated mitotic phenotypes. I consider this phenotypic rescue experiment is essential, and I agree with the authors about the revision plan to discern this issue.

We agree with the reviewer that the clarification on how microtubule-bundling activity of CCDC66 contributes to its mitotic functions is essential. As suggested by the reviewer, we performed new phenotypic rescue experiments and included results for 5 new phenotypic rescues in Fig. 7 and Fig. S7 of the revised manuscript. Specifically, we performed rescue experiments for K-fiber integrity, spindle positioning, central spindle intensity and organization, and cytokinesis defects to distinguish between centrosomal and non-centrosomal microtubule nucleation as well as MT organization activities of CCDC66. We performed these experiments in cells stably expressing mNG, mNG-CCDC66, mNG-CCDC66 (570-948) and mNG-CCDC66-PACT. Below is the summary of these experiments:

E) In Fig. 7E, we plotted **central spindle microtubule intensity** of the stable cell lines transfected with control or CCDC66 siRNA. Expression of mNG-CCDC66 and mNG-CCDC66 (570-948) restored the decrease in central spindle microtubule intensity associated with CCDC66 depletion. The centrosome-restricted mNG-CCDC66-PACT did not restore this phenotype. This experiment together with nocodazole washout experiment in new Figure 6B, where we identified defects in acentrosomal microtubule asters, provide strong evidence that CCDC66 is involved in non-

centrosomal microtubule nucleation and through its microtubule binding activity. We further quantified rescues of **aberrant central spindle formation** in Figure S7F and again mNG-CCDC66 and mNG-CCDC66 (570-948) restored this defect while mNG-CCDC66-PACT did not, further supporting the role of CCDC66 microtubule bundling activity in organization of central spindle in anaphase.

F) In Fig. 7F, we plotted percentage of **binucleated cells** of the stable cell lines transfected with control or CCDC66 siRNA. Expression of mNG-CCDC66 fully and mNG-CCDC66 (570-948) partially restored the cytokinesis defect in CCDC66-depleted cells. However, mNG-CCDC66-PACT did not restore this phenotype, indicating that microtubule-binding and bundling activity of CCDC66 is required for its functions during cytokinesis. The partial rescue by mNG-CCDC66 (570-948) shows that its N-terminus also contributes to CCDC66 functions during cytokinesis. N-terminus might contribute to cytokinesis by binding to and regulating microtubules and/or by recruitment of cytokinetic factors to the spindle midzone. Taken together, these results together support that the microtubule bundling activity of CCDC66 is required for cytokinesis.

G) In Figure S7E, we plotted microtubule intensity at the **K-fibers** in cold-treated cells. Expression of mNG-CCDC66 and mNG-CCDC66 (570-948) both rescued these phenotypes. However, mNG-CCDC66-PACT did not. These results show that MT binding activity of CCDC66 is important for their stability and organization.

H) In Fig. S7C, we plotted the **spindle angle** of the stable cell lines transfected with control or CCDC66 siRNA. Expression of mNG-CCDC66, mNG-CCDC66 (570-948) and mNG-CCDC66-PACT all rescued the spindle positioning defects associated with CCDC66 depletion. The rescue by mNG-CCDC66-PACT can be explained by the increased microtubule nucleation from the spindle poles in part due to higher levels of gamma-tubulin. As for mNG-CCDC66 (570-948), it might contribute to spindle positioning via stabilizing microtubules. These results show that CCDC66 functions during spindle positioning by regulating centrosomal microtubule nucleation as well as stability/organization of astral microtubules

We now included the results from the new phenotypic rescue experiments in Fig. 7 and Fig. S7 and discussed the results in the discussion section in terms of their implications in our understanding of relative contributions of CCDC66 activities and cellular pools to its mitotic and cytokinetic functions.

As I mentioned in my previous revision, the results presented in this work are attractive to the general cell biological community, especially the centrosome and cilia communities. The authors are supported by solid evidence of their work, and their proposal strategy to address reviewers' comments is well conducted.

We are encouraged to see that the reviewer found our study of general interest and highlighted that our conclusions are supported by strong data and our revision strategy is well conducted. We hope that the reviewer aggress that the phenotypic rescue experiments we performed with different CCDC66 mutants strengthens our conclusions on the regulation of mitosis and cytokinesis by CCDC66 via promoting microtubule nucleation and organization.

Reviewer#4:

In this manuscript the authors study the protein CCDC66 that was previously found to be localized on centrosome and MT and to be involved in mitotic spindle assembly (by the same team and others). Here the authors try to understand how CCDC66 participates to spindle organization.

1- Although they found that CCDC66 is required to localize centrosome proteins important for spindle assembly (centrosome maturation) it remains puzzling to understand how it works. CCDC66 is also a MAP that bundles MT, and it is also as puzzling to reconcile these two functions. The manuscript is very confusing, the experiments are well done but often one does not understand their choice and the order in which they are done. The results are often over-interpreted, sometimes even very badly interpreted. The manuscript shows interesting results but too preliminary as it is.

Regarding the criticism that the reviewer raises on the order of the data presented in the manuscript, we evaluated it together with the comments we received from the other three reviewers and reorganized the revised

manuscript using the following logical flow: 1) CCDC66 localization during cell division, 2) CCDC66 interactions during cell division, 3 and 4) defects associated with CCDC66 depletion in early mitosis, 5) defects associated with CCDC66 depletion in later stages of mitosis and cytokinesis 6) CCDC66's roles during centrosomal and non-centrosomal microtubule nucleation in mitosis, 7) Phenotypic rescue experiments.

In our manuscript, we combined localization, interaction, loss-of-function and phenotypic rescue experiments and uncovered and reported for the first time the non-ciliary functions and mechanisms of the ciliopathy protein CCDC66. To highlight these points, we included a model figure in Fig. 8 and also summarized below the main points of our work:
1) We showed that CCDC66 dynamically localizes to the spindle poles and the bipolar spindle in metaphase, central spindle in anaphase and midbody in cytokinesis. In agreement with its localization, we showed that CCDC66 interacts with core pericentriolar material proteins implicated in centrosome maturation and spindle MAPs required for MT nucleation and organization.

2) CCDC66 is required for mitotic progression via regulation of spindle assembly, organization and positioning, levels of spindle MTs, k-fiber integrity and chromosome congression. Moreover, CCDC66 functions during cytokinesis in part by regulating assembly and organization of central spindle and midbody MTs. Our results revealed MT nucleation and organization as the two major mechanisms by which CCDC66 functions during cell division.

3) CCDC66 is required for spindle orientation and positioning. Importantly, we showed that CCDC66 mediates these functions by regulating astral microtubule density and length.

4) CCDC66 regulates the assembly, stability and/or organization of k-fibers as well as spindle and midbody microtubules. Using cellular and *in vitro* MT pelleting and bundling assays, we identified CCDC66 as a MAP that crosslinks MTs. Our results identify CCDC66 as a new MT-crosslinking MAP required for organization of microtubule bundles during cell division.

5) Using cells expressing mNG-fusions CCDC66, CCDC66 (570-948) and CCDC66-PACT, we performed rescue experiments for 7 processes impaired upon CCDC66 depletion, which include spindle microtubule density in metaphase, gamma-tubulin recruitment to spindle poles, k-fiber stability, central spindle microtubule intensity and organization, spindle positioning and cytokinesis. The results of these experiments showed that CCDC66 functions during mitosis and cytokinesis via regulating centrosomal and non-centrosomal MT nucleation as well as MT organization. Importantly, the relative contribution of these activities to different CCDC66 functions varies based on the mechanisms by which different MT arrays are assembled and organized.

Based on the comments from 4 reviewers, we have extensively revised the manuscript and addressed the major and minor concerns. The revised version includes significant new results that strengthen our conclusions and even more importantly reveal the mechanisms by which CCDC66 mediates its functions at the centrosomes and microtubules. Additionally, the data presented in the revised manuscript better highlights the broader impact of our conclusions as they pertain to understanding the relationship between the pleiotropic CCDC66 functions during cell division. We hope that the reviewer agrees and acknowledges the advances that our manuscript provides to the field.

2- CCDC66 seems to be involved in the nucleation of MTs from centrosomes. Without CCDC66 the bipolar spindle is formed, this spindle has a normal length but the global network of mitotic MTs is less dense. This is interesting but how does it work? There is no mechanism described that would explain this phenotype.

As the reviewer noted, we did not find a difference in spindle length upon the CCDC66 depletion although there is significant defect in spindle MT levels via regulating centrosomal and non-centrosomal MT nucleation. Centrosomal MT nucleation is not one of the three critical mechanisms determining the spindle length, which are: MT polymerization, sliding and depolymerization (Goshima et al., 2005 - PMID: 16303556; Bird and Hyman, 2008 – PMID: 18663142; Dumont and Mitchison, 2009 - PMID: 19906577). It is quite possible that depletion of CCDC66 did not perturb other MT polymerizes, and kinesins that affect MT sliding and depolymerization, which might then compensate for spindle length defects. We now revised the related discussion section for this phenotype in pg. 25 as follows:

“*Despite its roles in MT nucleation and organization, CCDC66 depletion did not result in shorter spindles, which can be explained by a compensatory mechanism activated in CCDC66-depleted cells. Spatiotemporal regulation of MT polymerization, depolymerization, and sliding is critical to spindle length maintenance, providing remarkable ability of metaphase spindles to correct transient fluctuations in morphology (Goshima et al., 2005; Dumont and Mitchison, 2009). Other MAPs and molecular motors that regulate MT stability, dynamics, sliding, as well as regulators of chromatid cohesion and chromosome MT nucleation (Bird and Hyman, 2008 - PMID: 18663142), probably compensate and correct MT perturbation in CCDC66 absence to maintain steady-state spindle length. Further characterization of the functional relationship between CCDC66 and MAPs in its proximity interactome, identification of the CCDC66 mitotic interactome and in vitro MT reconstitution assays will contribute to better understanding of regulation of spindle properties by CCDC66.”*

2- Also, the controls are not shown in the right places. Two examples (1) siRNA depletion should appear to control for the specificity of the localization observed with the anti-CCDC66 antibody (2) rescue should demonstrate the specificity of the observed phenotype. However, the localization (fig1) is associated with a long discussion/interpretation on the possible function of CCDC66 while the control (siRNA) is only shown in figure S3.
Same for the depletion (fig 4) which is very much discussed and interpreted while the control rescue is only shown fig 7.

As the reviewer suggested, the validation of the CCDC66 antibody by siRNA experiments and specificity of the CCDC66 loss-of-function phenotypes could have been introduced earlier in the manuscript. Figure 1 and S1 are limited in space due to the extent of data we included for CCDC66 localization in two different cell types. Therefore, space limitation is a problem in including the siRNA and rescue data in Figure 1. Additionally, we chose to include this data later in the manuscript for the following reasons:
- Fig. 1 and Fig. S1 includes data on localization and dynamics of CCDC66 and its truncations during cell division in two different cell lines. We chose to include this data in Fig. 3 when we first use siRNA-mediated loss-of-function experiments to study precise roles and mechanisms of CCDC66 during cell division. Loss of CCDC66 signal in immunofluorescence and immunoblotting experiments we included in Fig. S3 confirms efficient depletion of CCDC66 and validates our approach. Moreover, as the reviewer highlighted, these results also confirm the specificity of the endogenous CCDC66 localization.
- We performed phenotypic rescue experiments with mNG-CCDC66, mNG-CCDC66 (570-948) and mNG-CCDC66-PACT. While the results from the mNG-CCDC66 confirm the specificity of the phenotypes analyzed, the results from the latter two fusion proteins allowed to distinguish between the functions of centrosome and microtubule-associated pools and activities of CCDC66. Taken together, the four different phenotypic rescue experiments we performed with three different fusion proteins provide mechanistic insights into CCDC66 functions. Therefore, we included this data as a separate figure where we dissect mechanisms that underlie the reported functions.

As suggested by the reviewer, we now revised the results section for Fig. 1 and S1 to make it more concise by removing technical details and discussion of data.

3- The manuscript must be rewritten in a more logical way and progression and additional experiment performed to propose a mechanism to explain the function of CCDC66.

In our rebuttal to point 1 of the reviewer’s concern, we summarized the main findings of our work regarding the functions and molecular mechanism of action of CCDC66 during cell division. By combining localization, interaction, loss-of-function and phenotypic rescue experiments, we for the first time report the non-ciliary functions and mechanisms of CCDC66. We hope that the reviewer agrees with the advances our manuscript provides to the field.

Regarding the flow of our manuscript, we reorganized it using the following logical flow: 1) CCDC66 localization during cell division, 2) CCDC66 interactions during cell division, 3 and 4) defects associated with CCDC66 depletion in early mitosis, 5) defects associated with CCDC66 depletion in later stages of mitosis and cytokinesis 6) CCDC66’s roles during centrosomal and non-centrosomal microtubule nucleation in mitosis, 7) Phenotypic rescue experiments.

4- The manuscript also needs proofreading and correction of English.

Before the submission, we had our manuscript proof-read by members of my laboratory as well as several other principal investigators and corrected the minor grammatical errors we had identified.

Specific comments

1- Page 8 the authors say
"Like other PCM proteins, CCDC66 formed resolvable rings at the PCM (Fig. 2A, 2B)"
I do not see any ring of CCDC66 on the spindle poles. May be in prometaphase but the resolution is not good enough to conclude.

We agree with the reviewer that the resolution of the microscope we used for imaging CCDC66 localization during cell division is not sufficient to resolve whether CCDC66 forms rings at the PCM (except for Fig. 2C). To avoid over-conclusion, we removed this sentence and only concluded that CCDC66 localizes at the PCM.

2- Page 9
Is this sentence correct?
CCDC66 co-pelleted with myc-BirA* fusions of CDK5RAP2, Cep192, Cep152 and gamma-tubulin, but with the negative control myc-BirA* (Fig. 2E). I think it should be but NOT with the negative control myc-BirA

We corrected this sentence.

3- Page 9 again
"FLAGminiTurbo did not co-pellet with myc-BirA* fusions of these positive interactions, and CCDC66 also did not co-pellet with the MT plus-end-tracking protein EB1, confirming the specificity of its interactions with PCM proteins (Fig. 2E)". One cannot draw such conclusion: not co-pelleting with EB1 is not enough to confirm the specificity of the interaction with the PCM proteins, it might only "suggest"

For the co-immunoprecipitation experiments we performed in Fig. 2E, F, we used several different controls:
- We used Flag-miniTurbo empty vector to test whether the select proteins bind to the tag fused to CCDC66.
- The expression vector we used to test whether CCDC66 interacts with gamma-tubulin had T2A between gamma-tubulin and EB1. By examining the presence of these two proteins in the eluates from CCDC66 pulldown, we concluded that CCDC66 interacts with gamma-tubulin, but not EB1. Given that EB1 is also a MAP and does not interact with CCDC66, this result increases the confidence of our conclusions on the specificity of the interactions we identified for CCDC66 in Fig. 2. To avoid overconclusion, we removed the part of the sentence that states "…confirming the specificity of its interactions with PCM proteins."

4- Page 9 & 10
"Taken together, our results suggest that CCDC66 functions during mitosis and cytokinesis by regulating centrosome maturation, MT nucleation and/or organization"
This is an overinterpretation, I would rather write:
"Taken together, the localization of CCDC66 during mitosis and cytokinesis led us to test potential functions of CCDC66 in the regulation of centrosome maturation, MT nucleation and/or organization"

We revised the sentence as suggested by the reviewer.

5- Control
I would put the figure S3B in figure 1 as a control of the specificity of the antibodies used in fig1A (at least figure S3A and S3B in figure S1) this would reinforce the initial data.

We agree with the reviewer that Fig. 1 or S1 can be a better place for confirming the specificity of the CCDC66 antibody. However, both Fig. 1 and S1 is very dense due to localization and dynamic behavior data in two different cell lines. Therefore, moving Fig. S3A and 3B in these figures is not possible due to space limitations. Moreover, the localization and expression data for CCDC66 in cells transfected with control and CCDC66 siRNAs confirms efficient depletion of CCDC66, which is important for interpretation of the loss-of-function experiments.

6- Page 11 Microtubule densities after CCDC66 siRNA
"In agreement, we noted that the MT arrays of the bipolar spindle, central spindle and midbody of CCDC66-

depleted cells were disorganized and prominent defects in the assembly and organization of the bipolar spindle, central spindle and midbody such as disorganized MTs AND REDUCED MT INTENSITIES (Fig. 3E, S3D)." positioning. Relative to control cells, CCDC66
"… depletion resulted in a minor decrease in average spindle lengths, which was measured as pole-to-pole distance in metaphase cells (Fig. 4A)."
I disagree, I don't see any difference

We agree with the reviewer. In fact, our analysis of the spindle length phenotypes was a concern raised by three different reviewers. As noted by the reviewers, the defect we reported was significant but was very minor (0.935-fold).

As suggested by the second reviewer, we reanalyzed our raw images to take into account the impact of the tilt angle of the spindles on spindle length for Fig. X. $\alpha=180*\tan-1(h/L)/\pi$ is the formula we used to calculate spindle angle where h represents the stack difference between two spindle poles and L represents the distance between spindle poles when projected to the same stack. Therefore, this formula allowed us to analyze spindle length by taking into account the tilt angle of spindles.

The original analysis we performed for spindle length revealed a 0.935-fold decrease in spindle length upon spindle length. However, the results from the reanalysis showed that CCDC66 depletion does not results in a significant spindle length phenotype. In the light of these results, we changed our conclusions in the revised manuscript and removed the rescue experiments for the spindle length phenotype.

7- Page 12
"The tubulin fluorescence intensity at the spindle decreased about 0.6-fold in CCDC66-depleted cells relative to control cells (Fig. 4B, 4C)."
This is pretty obvious and this is the phenotype the authors must concentrate on.

We agree with the reviewer. This is the phenotype that led us to investigate the role of CCDC66 during centrosomal and non-centrosomal microtubule nucleation in Fig. 6. Together with the interactions we identified between CCDC66 and PCM proteins, these experiments identified CCDC66 as a regulator of centrosomal and non-centrosomal microtubule nucleation during mitosis. These functions explain why MT levels at the spindle is reduced upon CCDC66 depletion.

8- Page 12
Fig 4B it appears that the whole MT network is less dense, MT nucleation or stability is affected. It seems that CCDC66 siRNA affect all MT not specifically Astral MT of K fibers.
"This result identifies CCDC66 as a regulator of K-fiber integrity, which explains the chromosome alignment defects and mitotic failure observed in CCDC66-depleted cells (Fig. 3)."
This looks again like an overinterpretation

We agree with the reviewer that CCDC66 operates at different microtubules during mitosis. We specifically included this conclusion for Fig. 4D as it tests the stability of k-fibers. Our other data and figures comment on other types of microtubules being affected by CCDC66 depletion, which supports other phenotypes as spindle orientation defects and cytokinesis, but only destabilized K-fibers could support chromosome misalignment that we see in CCDC66 depletion.

9- Fig 5E, F and G
The images show a huge difference in the intensity of the centrosome labelling of gamma tub, pericentrin and CDK5RAP2 (IN PARTICULAR CDK5RAP2), whereas the data that represent mean ±SEM of two (Pericentrin) and three (gamma-tubulin, CDK5RAP2) independent experiments show very little difference. A comment might be necessary to say that the authors selected the best picture to illustrate the difference.

We now changed the representative images so that they reflect the average fold change identified over experimental replicates.

10- Fig S5
"Notably, CCDC66-depleted cells had an increased number of MT "nucleating centers than control cells, suggesting possible activation of non-centrosomal MT nucleation pathways (Fig. S5A)"

This is a pretty good example demonstrating that numbers are useful, looking to the figure S5A one can find only one more nucleation point in cells depleted of CCDC66. I am not sure that it is a significant increase. However, the intensity of gamma tub labelling of each point increases in control cells but do not increase in CCDC66 depleted cells. This rather tells that nucleation of MT is affected.
I would ask the authors to measure the intensity of gamma tub labelling of each point and compare with and without CCDC66.

To distinguish between centrosomal and non-centrosomal nucleation sites after nocodazole washout, we stained cells with centrin (centrosomal) instead of gamma-tubulin in new sets of experiments. We performed two different analyses in these cells. First, to assess their microtubule nucleation capacity, we quantified the microtubule aster size by drawing a freehand shape around the edges of the microtubules at the aster and quantified the area. The microtubule aster size at the centrosomal and non-centrosomal nucleation centers were reduced upon CCDC66 depletion at 3, 5 and 8 min after nocodazole washout (Fig. 6B). Second, we quantified the number of non-centrosomal nucleation sites, which were higher in CCDC66-depleted cells as compared to control cells (Fig. S6A). This suggests that CCDC66 loss might activate non-centrosomal MT nucleation.

We also quantified the intensity of gamma tubulin both at the spindle and spindle microtubules, which revealed a significant decrease (Fig. 6E). Taken together with its interaction with gamma-tubulin, these results suggest that CCDC66 regulates microtubule nucleation in part via gamma-tubulin recruitment.

11- Fig 6 and page 14
"During the analysis of CCDC66-depleted cells stained for MTs, we noted that the cleavage furrow ingression is highly asymmetric and/or skewed in a significant number of cells (Fig. 6C)."

highly asymmetric and/or skewed?
The authors must be more precise

We used the word asymmetric because this refers to ingression furrow, which as the reviewer pointed out the furrow might be defective, and we are aware of this. Spindle might be skewed (bent) not purely due to the defects in MT organization but because the membrane is pushing from one side more than the other. We do not have a membrane marker therefore we are careful and less precise allowing for both possibilities by using words and/or. The word skewed we used is more precise than bent and includes any bending, twisting and curving in cytokinetic bridge MT and precisely describes observation by IF (from Cambridge Dictionary)

The telophase cleavage furrow shown in Fig6C bottom is it asymmetric, skewed, bent …? What is the % of such images …
Also it is difficult to draw any conclusion by only looking to and comparing the shape of the telophase cleavage furrow.
I invite the authors to read the following paper (Lafaurie-Janvore J, Maiuri P, Wang I, Pinot M, Manneville JB, Betz T, Balland M, Piel M. ESCRT-III assembly and cytokinetic abscission are induced by tension release in the intercellular bridge. Science. 2013 Mar 29;339(6127):1625-9)

We thank the reviewer for recommending the Lafaurie-Janvore et al. 2013 paper that quantified abscission time using quantitative laser ablation, micropattern and dynamic imaging experiments. We carefully went through quantitative approaches the authors used to characterize the effect of bridge tension and ESCRT-III on the abscission time. However, the nature of experiment they used for data analysis is very different from the ones we generated and therefore we could not adapt their methods.

Instead, we adapted the methods and terminology used in the Fig. 4 of the Taulet et al. Nature Comm 2017 paper (PMID: 29203870) for elucidating the role of IFT proteins in controlling the geometry of cleavage furrow ingression. Specifically, we quantified the percentage of cells with asymmetric cleavage furrow ingression. As compared to control cells, cleavage furrow ingression were highly asymmetric in a significant number of CCDC66-depleted cells (siControl: %23.79, siCCDC66:%63.85). This result show that CCDC66 is required for proper organization of the cytokinetic bridge.

We used the word asymmetric because this refers to ingression furrow, which as the reviewer pointed out the furrow might be defective, and we are aware of this. Spindle might be skewed (bent) not purely due to the defects in MT organization but because the membrane is pushing from one side more than the other. We do not

have a membrane marker therefore we are careful and less precise allowing for both possibilities by using words and/or. The word skewed we used is more precise than bent and includes any bending, twisting and curving in cytokinetic bridge MT and precisely describes observation by IF.

12- The rescue
These are very nice experiments, that strengthen the data
CCDC66-PACT rescue MT densities, this is surprising that the only localization at the centrosome is enough to rescue
570-948 also works but the protein is all over the place

In cells overexpressing mNG-CCDC66 (570-948), the fusion protein is mostly cytoplasmic. For quantification of phenotypic rescue experiments, we included the cells in which the fusion protein localizes to spindle poles and microtubules. Although we included another image that represents this localization, we also note that mNG-CCDC66 (570-948) has a higher cytoplasmic / spindle pool ratio as compared to the full length CCDC66.

13- I would like to see WB showing the level of expression of the different proteins (NG CCDC66 1-948) (NG CCDC66-PACT) & (NG CCDC66 570-948) in the rescue experiments.

We included two blots confirming the expression of the mNG fusions of CCDC66, CCDC66 (570-948) and CCDC66-PACT in cells transfected with CCDC66 siRNA. The blots in Fig. S7B together with the immunofluorescence data (Fig. 7) confirm that the fusion proteins are expressed at the right size and are resistant to CCDC66 siRNA. Although the western blot data indicates overexpression of the fusion proteins relative to endogenous protein, this does not reflect the expression level of the fusions in individual cells. The stable lines we used for rescue experiments were not 100% homogenous in terms of the expression levels of the fusion proteins. For quantification of the rescue experiments, we included the cells in which the fusion proteins were expressed at lower levels and localized properly to centrosomes and/or microtubules (representative images in Fig. 7). We revised the related methods section to detail how we performed quantification. It is of importance to note that stable mNG-CCDC66 and CCDC66 (570-948) cell lines are exhibiting lower levels of CCDC66 on MTs after longer time in cell culture due to the toxic effect of bundling and over-stabilization of MTs on cell division.

14- « We performed rescue experiments for defective targeting of gamma-tubulin to the spindle poles, reduced spindle tubulin intensity and SHORTER SPINDLE LENGTH associated with CCDC66 loss »
Well as I said in fig4A there is no detectable differences in the length of the spindle with or without CCDC66

As explained in response to point 6 of the reviewer, we reanalyzed the spindle length data by taking into account the impact of the tilt angle of the spindles on spindle length. The results from the reanalysis showed that CCDC66 depletion does not results in a significant spindle length phenotype. In the light of these results, we changed our conclusions in the revised manuscript and removed the rescue experiments for the spindle length phenotype.
