# Supplementary material for: The ciliopathy protein CCDC66 controls mitotic progression and cytokinesis by promoting microtubule nucleation and organization
Source: PLoS Biol. 2022 Jul 18;20(7):e3001708. doi: 10.1371/journal.pbio.3001708 (PMC9333452; doi:10.1371/journal.pbio.3001708)
Supplement: Attachment — Submitted filename: PointByPointResponse_SecondRound_final.pdf [file pbio.3001708.s021.pdf]

We thank all the reviewers the positive assessment of the revised manuscript. We have now addressed the further concerns raised by the reviewer#1 as explained bellow.

Reviewer #1:

This revised manuscript has been substantially improved. The points raised in the initial review have been addressed.

Comments:

1. For the assay of kinetochore fiber stability, the authors used a brief cold treatment to disassemble the non-kinetochore associated microtubules. Cells at metaphase have k-fiber microtubules that resist disassembly; cells prior to metaphase do not. Did the authors include MG132 to arrest cells at metaphase, thus avoiding the possibility that prometaphase cells are included in the analysis?

The reviewer raises a very good point regarding the differences in the stability of k-fiber microtubules at different stages of mitosis. In k-fiber stability experiments, we did not use proteasome inhibitor, MG132, to synchronize the cells at metaphase prior to cold treatment. The proteasome inhibitor treatment was similarly omitted in several studies by Royle et al., 2005 (PMID: 15858577), Archinti et al., 2010 (PMID: 20736305), Nixon et al., 2015 (PMID: 26090906), Ohta et al., 2015 (PMID: 25657325), Zhou et al., 2015 (PMID: 31521166). However, we are certain that we included only metaphase cells in our analysis, as we selected the quantified control and CCDC66-depleted cells based on chromosome congression at the equatorial plate. Our observation of chromosome alignment defects in CCDC66 (Figure 3H, 3I) supports mild effect and the wider metaphase plate, therefore, CCDC66-depleted cells did not significantly differ in their chromosome congression state compared to the control treatment in our cold assays.

Since the cold treatment disassemble interpolar and astral microtubules, as well as inhibit both the formation of new microtubules and the elongation of pre-existing microtubules (Reider, 1981; Rieder and Borisy, 1981), we are confident that we measured stability of only cold-stable kinetochore fibers in both treatments and rescue experiments. Importantly, the control and CCDC66-depleted cells were undergoing identical treatment and numbered of analyzed cells in all treatments was high (sixty-seven cells for siControl and eighty-one cells for siCCDC66 in Fig. 4E and Fig. S7E). We included the following sentences to Method section on pg. 35 to clarify how we quantified k-fiber stability:

"For k-fiber stability assay, control and CCDC66-depleted cells were placed on ice (4°C) for 10 minutes without previous treatment and cell synchronization with proteasome inhibitor MG132. Cells were washed extensively with cold PBS to prevent MT polymerization, then fixed with ice cold methanol at - 20 °C for 3 minutes. The tubulin fluorescence intensity of cold-stable K-fibers was measured as described above for quantification of spindle microtubule intensity."

2. What is the role of the N-terminal portion of CCDC66? The authors might state if there is a known role, as a reminder for the reader.

Previously, we observed that the N-terminal 570 amino acids, containing the CCDC66 conserved region localized to the centrosomes and decorated the microtubule network in cells similarly to the C-terminus (Conkar et al., JCS 2017, Figure 3, S3). Apart from the microtubule binding of this domain in interphase and cell division, we do not know whether this fragment binds to microtubules directly and if so, whether it affects microtubule organization. Since the partial rescue of increased binucleation by C-terminal region of CCDC66 suggests contribution of N-terminus to cytokinesis functions, we now included the following sentences on our previous results and the possible role of the N-terminus in the results and discussion sections on pages 9 and 24. We also highlighted in the results section that the C-terminal region partially rescues the mitotic and cytokinetic phenotypes.

"Previously, we showed that CCDC66 and its N-terminal 1-570 and C-terminal 570-948 amino acid residue fragments localize to MTs in cells (48). Given that C-terminal fragment binds to MTs directly, we ……."

“Further studies aimed at addressing how CCDC66 works together with PRC1 and other components of the central spindle and midbody, the factors contributing to contractile actomyosin ring (AMR) formation as well as elucidating the relative contribution of the N-terminal region of CCDC66 to its functions will be critical in providing mechanistic insight into CCDC66 functions during cytokinesis.”

3. Mispositioning and angle. The authors measure spindle tilt, a feature that they noticed was altered in the siRNA depleted cells. In the text they use "mispositioning" in several places to refer to this phenotype. Some cells position their spindle to one side of the cell, but to my knowledge the cells used in this study have centrally positioned spindles and the position of the spindle relative to the cell cortex was not actually measured. I think the term mis/positioning should not be used unless the authors mean that the spindle is off-center in a cell with a centrally located spindle. The feature measured was the angle relative to the substrate.

As the reviewer noted, we did not measure spindle position relative to the cell cortex. Although we noticed some instability in spindle position together with changes in spindle orientation in cells depleted of CCDC66 during the live cell imaging (Movies 3, 5, 6 and 7), due to the lack of cortical marker we measured only the change in the spindle angle compared to the substratum. Therefore, we replaced words “positioning” and “mispositioning” throughout the text with “position” and “misorientation”.

4. Centrosome and spindle pole. I realize that these two terms are commonly used interchangeably, but they are not actually the same. The centrosome, consisting of the centrioles and PCM, localizes at the spindle pole for spindles in cells that have centrosomes. Spindle microtubules converge into the spindle pole and the components localized there are not centrosomal components. see: 10.1242/jcs.111.11.1477

On the top of page 9, the authors say that CCDC66 is at the centrosome in interphase and at the spindle pole and microtubule-based structures in mitosis. In this instance, "at the centrosome" might be more accurate. A bit later, they describe results using hi-resolution imaging to localize CCDC66 relative to markers of the "spindle pole" (top of page 10) and go on to say they used antibodies to PCM proteins. The first sentence in this section should read "centrosome" or mitotic centrosome, because that is what they are looking at. This section should be edited to refer to the centrosome.

We agree with the reviewer that using centrosomes instead of spindle poles at times when we refer to centrosomal-microtubule nucleation is more specific. Therefore, we have replaced “spindle pole” with “centrosomes” in the specific places the reviewer identified as well as in other parts of the manuscript where we specifically refer to mitotic centrosomes.

The presentation of the data and the writing have improved the manuscript.
